# Twitter, time and emotions

**DOI:** 10.1098/rsos.201900

**Published:** 2021-05-26

**Authors:** Eric Mayor, Lucas M. Bietti

**Affiliations:** ^1^ Institute of Work and Organizational Psychology, University of Neuchâtel, Rue Emile Argand 11, Neuchâtel 2000, Switzerland; ^2^ Division of Clinical Psychology and epidemiology, Department of Psychology, University of Basel, MIssionsstrasse 61a, Basel 4055, Switzerland; ^3^ Department of Psychology, Norwegian University of Science and Technology, Dragvoll Campus, Trondheim 7491, Norway

**Keywords:** positive emotions, negative emotions, Twitter, circadian rhythms, circaseptan rhythms

## Abstract

The study of temporal trajectories of emotions shared in tweets has shown that both positive and negative emotions follow nonlinear circadian (24 h) and circaseptan (7-day) patterns. But to this point, such findings could be instrument-dependent as they rely exclusively on coding using the Linguistic Inquiry Word Count. Further, research has shown that self-referential content has higher relevance and meaning for individuals, compared with other types of content. Investigating the specificity of self-referential material in temporal patterns of emotional expression in tweets is of interest, but current research is based upon generic textual productions. The temporal variations of emotions shared in tweets through emojis have not been compared to textual analyses to date. This study hence focuses on several comparisons: (i) between Self-referencing tweets versus Other topic tweets, (ii) between coding of textual productions versus coding of emojis, and finally (iii) between coding of textual productions using different sentiment analysis tools (the Linguistic Inquiry and Word Count—LIWC; the Valence Aware Dictionary and sEntiment Reasoner—VADER and the Hu Liu sentiment lexicon—Hu Liu). In a collection of more than 7 million Self-referencing and close to 18 million Other topic content-coded tweets, we identified that (i) similarities and differences in terms of shape and amplitude can be observed in temporal trajectories of expressed emotions between Self-referring and Other topic tweets, (ii) that all tools feature significant circadian and circaseptan patterns in both datasets but not always, and there is often a correspondence in the shape of circadian and circaseptan patterns, and finally (iii) that circadian and circaseptan patterns obtained from the coding of emotional expression in emojis sometimes depart from those of the textual analysis, indicating some complementarity in the use of both modes of expression. We discuss the implications of our findings from the perspective of the literature on emotions and well-being.

## 1. Introduction

The advent of social networking sites has radically altered the ways in which emotions are communicated. Social media provides researchers with the unprecedented opportunity to track, almost in real time, changes in expressed emotions on a large scale over time and space. This can be used as an indicator of the overall emotional state of the population, providing important information for the design and implementation of public health campaigns [1–3]. For instance, recent studies have shown that social media users' linguistic style constitutes a useful tool for measuring and predicting depression [4], transitions from mental health discourse to suicide [5], eating disorders [6] and the worsening of psychotic symptoms [7].

Circadian (24 h) rhythms are reflected in changes in humans’ physiology and behaviours at multiple levels, from the timing of cellular activities to the coordination of daily cycles of behaviour [8]. They might have evolved in response to environmental variations following alternations between light and dark cycles [9]. The mammalian ‘internal clock’ relies on fluctuations in body temperature, which controls circadian rhythms throughout the day [10]. Body temperature peaks during the day and reaches its lowest at night, controlling phases of higher activity versus rest [11]. Markers of circadian rhythms include melatonin and cortisol release [12]. Circadian rhythms enable humans to temporally reorganize and adjust metabolic [13] and physiological processes in interaction with behavioural activities [14]. Optimal sleep and wake patterns are dependent on circadian rhythms, and their disruption due to artificial lighting and other external factors (e.g. shift-work and jet-lag) has negative consequences for memory and cognitive performance [13].

Regular variations in mood, cognition and behaviour also vary over longer periods of time, notably in circaseptan (7-day) rhythms [15]. Research has consistently shown the existence of circaseptan patterns in humans. Seven-day patterns are associated with cultural traditions (e.g. Shabbat as the holy day of rest in the Hebraic 7-day week) and the cultural distinction between weekdays and weekends in modern societies [16] regulating social practices and behaviours [17]. However, the existence of circaseptan patterns in several animal species, including humans, informs about their endogenous rather than solely social and cultural origin. The weekend may enable humans to resynchronize circadian rhythms after increasing residual desynchronization due to the accumulation of chemical substances in the body over the workweek [18]. Thus, the modern 7-day week may represent a cultural adaptation for realizing endogenous chronobiological processes, such as recovery from work at the weekend [19].

Circaseptan rhythms were found in changes in blood pressure of women during pregnancy [20], and newborns' heart rate and body weight [21], body temperature [22], eating behaviours [23] and mood [24]. In male and female children, circaseptan cycles also coordinate variations in cognitive functions [25], and in adults, they regulate patterns in physical activity, heart rate fluctuations, night-time sleep duration, nutrition, core body temperature and changes in the immune systems [26]. Emotions are also subject to circadian and circaseptan cyclical variations. Studies on temporal patterns in emotions and mood have broadly employed two approaches: self-reports and textual analyses of spontaneous productions, notably in social media.

### 1.1. Self-report studies

Monk *et al*. [27] have shown that self-reported happiness and well-being were highest 4–6 h after waking, which was co-occurring with increases in both body temperature and cognitive ability. Additionally, Clark *et al*. [28] observed that positive affect (PA) and negative affect (NA) follow opposite trajectories throughout the day: PA followed a reversed U-shaped curve, at its lowest at the beginning and end of the day, whereas NA followed a U-shaped curve, at its lowest between noon and midnight. More recently, researchers have found that individuals tended to experience highest levels of NA around 10.00 and around 16.00, while PA was highest around noon and 20.00 [29].

Overall, results show that PA and NA vary throughout the day, but not necessarily in the same way across studies. Differences in results might be due to the relatively small samples used and the lack of sample inclusiveness [30]. Moreover, inconclusive findings could be related to differences in chronotypes (morning-type individuals versus evening-type individuals) in the tested samples. For instance, Miller *et al*. [31] showed that evening-type individuals manifested delayed PA phases and presented less amplitude compared to morning-type individuals. Diurnal affect variations have been reported in healthy and depressed populations but had a smaller amplitude in the latter [32]. Interestingly, while several studies have shown that eveningness is associated with greater depression and lower PA [14], the evidence supporting the association between eveningness and NA is scarce and mostly observed in clinical populations only [33].

Studies on circaseptan changes in affect also show some degree of disagreement. Using the PANAS [34], Cornélissen *et al*. [24] have found that PA and NA follow both circadian and circaseptan changes. They found PA to be lowest on Sundays and NA on Saturdays. Similar patterns were found in a large heterogenous sample where PA (NA) was highest (lowest) during the weekend than other weekdays [35]. Among these weekdays, individuals were in a better mood on Friday, and in a worse mood on Monday (non-retired individuals only). Somewhat different results were found using the POMS [36]: two studies reported that PA (vigour) was generally highest on Sundays, and NA (fatigue, depression, anger, anxiety) was lowest during the weekend. Vittengl & Holt [37] found that PA was lowest on Sunday and increased throughout the week from Monday to Saturday but reported no significant change in NA throughout the week. Larsen & Kasimatis [15] have shown that subjective well-being varies in a sinusoid manner over the week and has a peak on Saturday, but such changes are more pronounced in introverts compared to extroverts.

### 1.2. Social media studies

Twitter has been the social media platform used to conduct most of research on emotions expressed through social media channels. Temporal variations in the expression of emotions in Twitter have been studied to detect emotional contagion [38], change in public opinions [39], identify mental disorders [40], monitor public health concerns [41], measure population mood before, during and after natural disasters [42], detect voting preferences in elections [43], predict changes in the stock market [44] and to estimate the duration of positive and negative emotions as the effect of affect labelling (i.e. explicitly putting one's feeling into words) [44]. Fan *et al*. [45] analysed the evolution of emotional contents in tweets posted between 2006 and 2012. They collected tweets that conveyed Twitter users' emotional state using as search criteria tweets that included the expressions ‘I feel … ’, ‘I'm feeling … ’ or ‘I am feeling’. These were categorized as affect labelling tweets [45]. Afterwards, they analysed the emotional language of other tweets 6 h before and 6 h after the affect labelling tweets. Fan *et al*. found that affect labelling mitigated emotional intensity over time and that the emotions lasted approximately 1.5 h from beginning to end.

The number of studies examining circadian and circaseptan rhythms in expressed emotions has been limited. Automated sentiment analysis tools such as the Linguistic Inquiry and Word Count (LIWC) have been broadly employed to investigate psychologically relevant processes that are subject to cyclical variations in social media. Emotions have probably been the process most studied. LIWC allows coding a diversity of content categories from the text on a word-by-word basis. Using LIWC-coded tweets, Golder & Macy [30] found that both PA (category posemo) and NA (category negemo) were highest at midnight, then tended to decrease until 4.00 for PA and 6.00 for NA during the workweek, and until 8.00 during the weekend. PA increased sharply after this nightly drop and decreased between 9.00 and 17.00 to increase again until 6.00. The increase in NA was less marked but continued until midnight. These findings were supported by more recent studies [46]. Golder & Macy found that PA was highest and NA lowest during the weekend. The worst days in terms of both dimensions are Mondays, Tuesdays and Wednesdays. Wang *et al*. [47] showed that expressions of stress and NA in tweets presented their peaks on Mondays and gradually decreased towards Thursdays with a marked dip on Fridays. Dzogang *et al*. [46] used factor analysis of LIWC-coded tweets to obtain two main factors which they considered highly emotional (positive and negative emotions) and have found similar patterns.

### 1.3. Emoji use in the social media

Mobile phones supporting input and rendering of emoji characters enabled these to become increasingly popular [48] and be labelled as the fastest-growing language in the world [49]. Kaye *et al*. [50] conducted an online survey where they asked participants to reflect on their use of emoji in virtual platforms. They found that the emojis served to aid personal expression by establishing emotional tone and lighten mood and to reduce the ambiguity of the message. Recently, these results were conceptually replicated in a large online survey involving 1000 participants which also showed that the expression of emotions was the main reason behind their use [51]. Studies focusing on the use of emojis as devices of emotional expression have found that they were more present in positive rather than negative messages [52] and that facial emoji were the preferred type to express emotions [53]. Non-facial emoji were mostly used to communicate joy [48].

The increasing interest in the expression of emotions via emoji in social media also led to the development and validation of emoji sentiment lexica in various languages [54]. Numerous studies have investigated the use of emoji as a vehicle for the expression of emotion in social media. Zhao *et al*. [55] analysed 3.5 million Weibo messages that contained emoji conveying emotions. Emojis were classified into four categories of sentiments (angry, disgusting, joyful and sad) [55] examined hourly, weekly and monthly changes in the dataset. The authors found that people tend to be sad and angry from 6.00 to 8.00, but these emotions turned into joy after 10.00. Such a trend continued until the evening when sadness increased. Weekly patterns showed that people expressed increased joy towards the weekend with a peak on Saturdays. On Sundays, joy decreased and sadness and anger increased. Interestingly, Zhao *et al*. [55] observed that the expression of emotions in emoji on a monthly basis was highly dependent on local, national and international news and the region where the data was collected.

### 1.4. Self-reference

An important distinction in self-reference research is made between self-descriptions (e.g. mentions of states and traits) and autobiographical aspects (e.g. mentions of past events [56]). Both forms of self-reference are termed self-disclosure when addressed to others, for instance, in face-to-face interaction or through the social media [57].

Research on self-referencing processing has notably investigated whether self-reference is cognitively specific, i.e. distinct from the reference to other subjects or objects. Self-reference leads to deeper processing due to the higher interconnectedness of concepts related to the self, and has distinctive effects, such as memory facilitation due to motivational significance [58]. Indeed, seminal studies have shown that individuals recall better content that is related to themselves than content related to other targets [59].

More recent research has shown that self-reference integrates perceptual cues in memory [60]. Further, positive traits are better recalled than negative traits when the target is the self, but not if the target is someone else [61]; however, such effect vanishes in depressed individuals (negative self-schema; [62]). Individuals automatically allocate more attention to self-referential emotional cues than neutral cues [63]. Self-referential processing is also known to facilitate social cognition (e.g. empathic accuracy, theory of the mind; [64]). Referring negatively to the self increases the odds of depressive relapse [65]. This can be explained by the importance (of the valence) of self-references in predicting self-esteem and self-efficacy [66].

On the production side, the question of the effects of self-reference has mostly been studied in interaction (self-disclosure). The relevance of self-reference in social perception was already highlighted a few decades ago [67] and recent research has shown that it increases liking, notably when reciprocal [68]. As in face-to-face interaction [69], self-reference on social media promotes social worth, social support [70,71] and increases chances of friendship maintenance [72]. Further, honest and accurate self-reference in Web posts is linked to decreased loneliness [73]. Research has consistently shown that self-reference processing and production is distinct from references to other objects and topics and is highly significant to individuals. A recent study has shown reduced emotional intensity over time in Self-referencing tweets (in the ‘I am feeling’ form; [45]). However, no previous study has used Self-referencing tweets instead of generic tweets to examine circadian and circaseptan patterns of emotions expressed in tweets or have compared self-referencing with other topic productions in the study of emotions in social media [57].

### 1.5. The present study

Past research in circadian and circaseptan patterns of emotions on Twitter has been interested in generic (unfiltered) tweets, i.e. researchers did not distinguish between topics during or after data collection. But there is evidence that information relating to the self, as opposed to other topics, is better recalled, has an increased personal relevance and meaning for individuals and relates to different cognitive processes and emotional underpinnings [58,59,74,75]. Another potential shortcoming of past research in circadian and circaseptan emotional patterns has been the exclusive use of one coding tool, namely the LIWC [76]. Hence, the (in)dependence of obtained results upon this instrument remains to be investigated. Finally, past research on temporal emotional patterns has focused, almost exclusively, on textual productions in tweets. Whether emotional patterns in emojis are similar or display complementarity in the expression of emotions remains unknown. It is certainly of interest to investigate whether the general patterns of emotional expression found in tweets using the LIWC (the existence and shape of these patterns) hold in the specific case of the LIWC emotional categories only, or if similar findings can be obtained with other tools. In the latter case only could they be considered instrument-independent.

The present study proposes three major contributions. We compare patterns of change in emotions in self-referencing tweets (or *I am* tweets) and other topic tweets and thereby examine the distinctiveness of self-referencing tweets. We investigate differences in results obtained through the use of different sentiment analysis tools: the Linguistic Inquiry and Word Count (LIWC), the Valence Aware Dictionary and sEntiment Reasoner (VADER) and the Hu Liu sentiment lexicon (Hu Liu) (see Material and methods) and thereby assess the robustness of findings on circadian and circaseptan changes in the current literature. We then compare patterns of circadian and circaseptan change in emotional dimensions coded from emojis and in text in order to determine whether these are complementary or symmetrical. The sentiment analysis tools that we selected include both open source (e.g. VADER) and commercial (e.g. LIWC) instruments. The use of different instruments for the automatic coding of the same dataset is essential to assess the robustness of results across tools. Emotional expression in Twitter can not be fully captured relying upon textual analysis only. The decision to investigate emotional patterns in emojis extends the scope of such traditional, but maybe fractional research.

Finally, our study is the first to use mixed-model regression in order to partial out variance lying at the level of the user, for the study of circadian and circaseptan patterns of emotional variation. This allows an improved estimation of model parameters as well as of statistical significance and is innovative in the considered research area.

## 2. Material and methods

We used the R package *rtweet* to collect (i) Self-referencing tweets for four consecutive weeks through the Twitter Application Programming Interface. The search started on Monday, 3 September 2018, at 10.00 UTC and ended on Monday, 1 October 2018, at 10.00 UTC. We used the query "\"I am\" OR \"I\'m\" OR \"Im\"". (We included the verb *to be* in the present tense only to focus on present events and thoughts as much as possible—*I am going to* statements are future-oriented but match our search query as well.) We also collected (ii) generic tweets using the query ‘ ’, matching all tweets that contain a space. For these two queries, we searched for 100 tweets every 30 min (excluding retweets) in each of the 160 most populated US counties, of which the aggregate population represents more than half of the US population [77]. We used the counties' population centroid and square root of the radius of the area/Pi, according to the 2017 US Gazetteer Files [77], as centres and radiuses for the search within each county. We obtained a total of 7 577 640 Self-referencing tweets after discarding duplicate tweets based on status_id (tweet identifier), on average 70.47 per hour and county (s.d. = 26.52). These tweets were used in the textual analysis of Self-referencing tweets, whereas the analysis of emojis was based upon the subsample containing emojis (*N* = 1 182 477, 15.6%). We also obtained 18 367 569 generic tweets. From these, we created a pool of Other topic tweets (not Self-referencing) by excluding 500 600 tweets (2.8%) which matched the query for Self-referencing tweets. From these 17 866 969 Other topic tweets, we randomly sampled 7 577 640 in order to match the sample size of Self-referencing tweets. These were subjected to textual analyses. The analysis of emojis for Other topic tweets was also based on a random subsample of the tweets containing emojis in Other topic tweets (for the complete corpus: *N* = 2 365 947) matching the observations in the Self-referencing corpus (for the subset: *N* = 1 182 477).

The study relied on data publicly available at the moment of data collection. We, therefore, did not seek the approval of an ethical review board for this study. The tweets were made public by the users themselves, and their use complies with the developer licence granted by Twitter. We have made the coded data and the identifier of each tweet available on OSF: (https://osf.io/4c7kd/). Using the identifiers, the tweets can be downloaded directly from Twitter.

### 2.1. Measures

#### 2.1.1. Time

Retrieved tweets received a timestamp in the POSIX format corresponding to the UTC date and time of their post on Twitter. UTC time was converted to the local time of the county in which tweets originated, after which we computed Hour and Day as numeric variables. We used the American convention for the ordering of the days of the week (leading to values of 0 for Sunday and 6 for Saturday).

#### 2.1.2. LIWC dimensions

The LIWC 2007 English dictionary allows for coding texts along 64 categories by simple word count. Categories relate to: linguistic processes (e.g. types of pronouns, types of verbs, verb tense, prepositions, quantifiers); psychological processes, notably composed of social processes (mentions of family, friends, humans); affective processes (overall score of positive and negative emotions, specific negative emotion categories); cognitive processes (insight, causation, etc.), relativity (e.g. time and motion), personal concerns (e.g. money, leisure, and religion) and spoken categories (e.g. assent and fillers). The LIWC dictionary was derived from multi-study validation work in psychology with iterative improvements spanning over decades (see [76]). Using the R package *Quanteda*, we coded each tweet for the categories of the LIWC 2007 English dictionary (proportions). For this work, we only used the emotional categories affect, posemo and negemo which have attested reliability [76]. The LIWC was chosen because it is the most frequently used tool in the study of emotional circaseptan and circadian patterns and because one of the aims of this study is to compare results from other tools with the LIWC.

#### 2.1.3. VADER sentiment dimensions

The VADER scoring algorithm [78] has been developed specially for the analysis of social media texts. An interesting feature of the VADER scoring is that it is not only based upon a lexicon, but also rule-based, and thus can handle negations (*not good* scoring opposite to *good*) and lexical ambiguity, which the other mentioned tools cannot. Another interesting feature of VADER is that it can handle not only the polarity of the emotion of the coded words but also their intensity. The resulting categories are Compound (an index of document positivity), Positive, Negative and Neutral. The categories are coded using a dictionary derived from complex machine learning algorithms. The classification results in good metrics in machine learning tasks. The VADER was chosen because it has been built for textual analysis in social media, can handle negations and is described as a promising tool in the literature. We also use the VADER to include a neutral emotional expression dimension for the textual analyses as a comparison with the neutral dimension in the analyses of emojis.

#### 2.1.4. Hu and Liu sentiment dimensions

A frequently used instrument, the Hu & Liu lexicon [79], was developed for sentiment analysis of customer reviews. The resulting categories (lexicon-based) are Sentiment (an overall measure of positivity), Positive and Negative (good classification metrics in machine learning tasks). This tool has been chosen because it has almost exclusively been used in studies that do not focus on textual production in the social media.

#### 2.1.5. Emoji sentiment dimensions

We coded emojis relying on the emoji sentiment ranking from Kralj Novak *et al*. [54] who used human raters to assess sentiment in tweets. The resulting categories are Sentiment (an overall measure of positivity), Positive (the probability of the emoji to appear in a tweet coded as positive), Negative (the probability of the emoji to appear in a tweet coded as negative) and Neutral (the probability of the emoji to appear in a tweet coded as Neutral) with acceptable classification metrics.

LIWC and VADER have been the sentiment analysis tools most widely used in psychology, linguistics and computer science [80]. Hutto & Gilbert [78] argued VADER to be more sensitive to sentiment text in social media than LIWC. For example, VADER accounts for acronyms, initialisms, emoticons and slang, which are relevant lexical items for sentiment analysis of text [81]. The Hu Liu lexicon suffers from the same limitations as LIWC [79]. A systematic review of sentiment analysis tools [80] showed that VADER generated higher accuracy sentiment rankings than LIWC for data collected from Twitter.

#### 2.1.6. Additional variables

The following variables were computed in order to examine patterns in the frequency and proportion of Self-referencing tweets: *Frequency* (per county/week/day/hour) was computed as the sum of occurrences in the Self-referencing tweet dataset, whereas the *Proportion* of Self-referencing tweets was computed as the sum of Self-referencing tweets (per county/week/day/hour) divided by the sum of all collected tweets in the complete generic tweet dataset.

### 2.2. Preprocessing and data analysis

For the textual analyses: Tweets were preprocessed using the following procedure: @user_mentions, links, non-ASCII characters, digits, tabs and punctuation characters were replaced with a space. Multiple spaces were then replaced with a single space. Leading and tailing spaces were removed. For the analyses of emojis: emojis were extracted and stored in a separate dataset which was coded using the emoji sentiment ranking [54].

Statistical testing allows us to determine whether the patterns we describe qualitatively represent significant quantitative changes. Data were analysed in R using random intercepts mixed-effects model regression. This was necessary because of the nesting of tweets within users, i.e. users could provide several tweets thereby generating variance at the user-level that needed to be partialled out. Participant ID was used as a clustering variable and all proportions were centred within-county. This step allowed us to also partial out variance laying at the level of counties in our analyses, which has not been undertaken in previous related research. The analyses were adjusted for week—entered as a factor. Adjusting for the week allowed us to ensure that results were not affected by the period of the month in which the tweet was posted. Our dependent variables were regressed on these control variables and polynomial contrasts (linear to quantic, i.e. up to the power of five) of variables hour and day in order to avoid multicollinearity. We use the term *polynomials* to refer to positive exponents corresponding to linear, quadratic, cubic, quartic and quantic functions (first- to fifth-degree polynomials) of the independent variables day and hour.

This approach allows modelling of up to four turning points in the dependent variables as a function of the (polynomials of the) independent variables. This is relevant because examining only linear and quadratic relationships would not account for the complexity that could be observed in the data.

We used the default estimator (restricted maximum likelihood) and optimizer (bobyqa) in the analyses. We used the Bonferroni significance correction to correct for the multiple tests we performed. Models using proportions of frequencies as outcome variables rely on counts/computations of these variables at the county level.

All models converged successfully. The overall effect of polynomials of day and hour, on the different dependent variables, was estimated using the *F* statistic on the obtained models (Type II ANOVA with Satterthwaite adjustment of degrees of freedom—this procedure leads to degrees of freedom that can vary for each predictor within models). After the Bonferroni adjustment, performed separately for circadian and ciracseptan patterns, the critical significance thresholds are *p* < 0.00179 for the *F*-tests of overall significance (28 tests performed per type of pattern) and *p* < 0.000179 (280 tests performed per type of pattern) for the mixed-model regression coefficients and corresponding *F*-tests of individual trends (each polynomial of day and hour, i.e. the linear, quadratic, cubic, quartic and quintic trends). The regression coefficients are provided in the text for the sake of completeness and because their sign is used when describing the contribution of the polynomial trends to the overall temporal trajectories (see §3.3). They are not an indicator of the importance of the individual trend. This is notably due to the fact that the predictors are transformed in order to make the polynomials orthogonal. We, therefore, comment on the magnitudes of the polynomial trends based upon the individual *F*-values (see §3.3 for this as well).

## 3. Results

Table 1 presents the correlations between all textual dimensions in the county-centred coding of Self-referencing tweets (above the diagonal) and Other topic tweets (below the diagonal) at the non-aggregated level. Correlations with the coding of emojis are not presented in this table as we used a subsample of tweets for these analyses. Similar correlations can be observed in the coding of Self-referencing tweets and Other topic tweets, with a few exceptions, such as *r* (Hu Liu-Sentiment, LIWC-affect). The positive as well as negative dimensions of all tools are highly correlated with one another in Self-referencing tweets (positive dimension: min *r* = 0.648; negative dimension: min *r* = 0.668) and Other topic tweets (positive dimension: min *r* = 631; negative dimension: min *r* = 0.652). Correlations of this magnitude are generally considered indicative of measures assessing the same construct [82]. The Hu Liu overall sentiment measure is strongly correlated with the VADER compound measure in both datasets (in Self-referencing tweets: *r* = 0.627; in Other topic tweets: *r* = 0.619), but not with the LIWC-Affect dimension. This was expected as the LIWC-Affect dimension captures textual emotionality independently of polarity.

**Table 1.** Correlation between coded textual dimensions. Correlations for self-referencing are presented above the diagonal. Correlations for Other topic tweets are presented below the diagonal. All correlations are significant at *p* < 0.001, except for r(Hu Liu-sentiment, LIWC-affect) in Self-referencing tweets.

	1	2	3	4	5	6	7	8	9	10
1. Hu Liu-sentiment		0.543	−0.553	0.627	0.492	0.017	−0.524	0.002	0.417	−0.404
2. Hu Liu-positive	0.589		−0.142	0.418	0.648	−0.417	−0.152	0.39	0.685	−0.124
3. Hu Liu-negative	−0.516	−0.101		−0.413	−0.15	−0.454	0.709	0.401	−0.134	0.668
4. VADER-compound	0.619	0.374	−0.374		0.693	−0.043	−0.657	0.077	0.518	−0.41
5. VADER-positive	0.501	0.631	−0.115	0.663		−0.616	−0.268	0.524	0.845	−0.133
6. VADER-neutral	−0.124	−0.481	−0.342	−0.164	−0.745		−0.594	−0.785	−0.581	−0.48
7. VADER-negative	−0.472	−0.115	0.652	−0.624	−0.209	−0.494		0.425	−0.152	0.723
8. LIWC-affect	0.125	0.491	0.332	0.155	0.624	−0.771	0.32		0.636	0.653
9. LIWC-posemo	0.415	0.658	−0.091	0.447	0.821	−0.65	−0.116	0.785		−0.13
10. LIWC-negemo	−0.366	−0.079	0.595	−0.367	−0.103	−0.395	0.715	0.517	−0.086	

**Figure 1.**
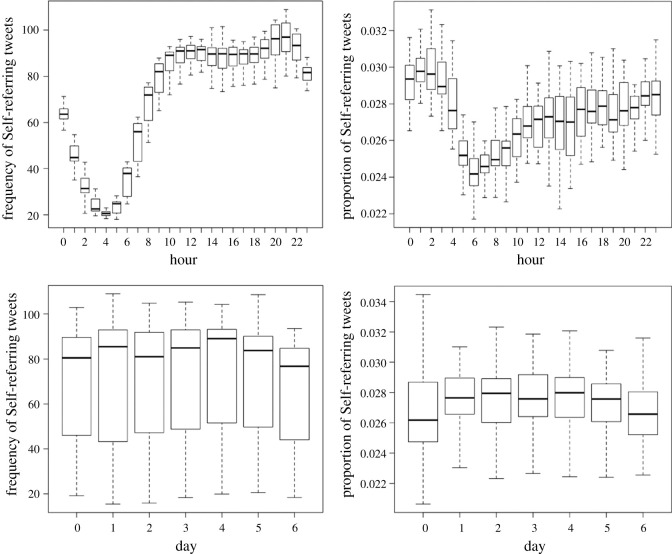
Observed frequency (per county) and proportion of Self-referencing tweets (upper half: circadian patterns; lower half: circaseptan patterns).

As shown in the upper half of figure 1, circadian patterns can be observed in the production of Self-referencing tweets (more tweets during the day, corresponding to wake–sleep cycles; left panel; Linear trend of Hour: *B* = 0.007, *p* < 0.0001; Quadratic trend: *B* = −0.002, *p* < 0.0001; Cubic trend: *B* = −0.003, *p* < 0.0001; Quartic trend: *B* = 0.003, *p* < 0.0001; Quintic trend: *B* = −0.002, *p* < 0.0001; *F*_5, 106 457_ = 18 779.67, *p* < 0.0001). The proportion of Self-referencing tweets follows a slightly different pattern: an increase throughout the day, with a minimum at 8.00 and a maximum between 2.00 and 3.00 (Linear trend of Hour: *B* = −0.008, *p* = 0.61; Quadratic trend: *B* = 0.38, *p* < 0.0001; Cubic trend: *B* = 0.20, *p* < 0.0001; Quartic trend: *B* = 0.005, *p* = 0.76; Quintic trend: *B* = 0.19, *p* < 0.0001; *F*_5, 106 820_ = 160.96, *p* < 0.0001).

Highest values in frequency can be observed at 20.00 and 21.00 and highest values in proportion at 1.00 and 2.00.

As shown in the lower half of figure 1, there was less variation throughout the week on these measures. Yet, these patterns are statistically significant (for frequencies: Linear trend of Day: *B* = −0.68, *p* = 0.011; Quadratic trend: *B* = −0.06, *p* < 0.0001; Cubic trend: *B* = −0.02, *p* < 0.0001; Quartic trend: *B* = −0.02, *p* < 0.0001; Quintic trend: *B* = 0.02, *p* < 0.0001; *F*_5, 106 820_ = 147.71, *p* < 0.0001; for proportions: Linear trend of Day: *B* = −0.003, *p* = 0.86; Quadratic trend: *B* = −0.14, *p* < 0.0001; Cubic trend: *B* = 0.20, *p* < 0.0001; Quartic trend: *B* = 0.005, *p* = 0.007; Quintic trend: *B* = 0.039, *p* = 0.03; *F*_5, 106 820_ =16.40, *p* < 0.0001). It is notable that the lowest frequency of Self-referencing tweets can be observed during the weekend and on Tuesday; the proportion of Self-referencing tweets being the lowest during the weekend. Both the frequency and proportion of Self-referencing tweets are highest on Thursday.

We hypothesized that the emotional dimensions of all considered instruments would vary as a function of polynomials of hour and day of the week in Self-referencing tweets and in Other topic tweets. The mixed-models regression overall results (*F*-values) are presented in table 2. We note the significant overall effect of polynomials of day and of hour for all dimensions in both datasets (at the Bonferroni-corrected *α* threshold). The degrees of freedom being close to identical in both datasets for each corresponding test, we compared the magnitude of the *F*-values using a difference corresponding to the minimal significant *F*-value (after the Bonferroni correction) as a threshold below which *F*-values would be considered identical. This only occurred in one case (positive dimension in emoji coding, circaseptan pattern), and in all other cases but one (LIWC-posemo dimension, circadian patterns), the discrepancy was much higher. This criterion was used because, to our knowledge, there exists no test allowing us to test the difference between *F*-values from mixed-model regression.

**Table 2.** Mixed-model regression results (ANOVAs). The minimum (7 038 460) and maximum (7 572 870) obtained denominator degrees of freedom for the textual tools were calculated using Satterthwaite's method. The minimum (1 135 066) and maximum (1 174 644) degree of freedom for emoji coding were calculated using the same method. All tests included five numerator degrees of freedom. All *p*s < 0.0004 (hence significant after the Bonferroni correction). For each comparison between datasets, the dimension with the highest value is indicated in bold (with a difference threshold corresponding to the minimal significant *F*-value after the Bonferroni correction).

	Self-referencing tweets			Other tweets	
	polynomials of day (IVs)	polynomials of hour (IVs)		polynomials of day (IVs)	polynomials of hour (IVs)
	*F*	*F*		*F*	*F*
LIWC			LIWC		
affect	184.26	**1017.37**	affect	**207.928**	741.72
positive	20.997	107.754	positive	**65.812**	**118.501**
negative	**242.33**	1437.714	negative	202.487	**1591.792**
HU LIU			HU LIU		
sentiment	**67.91**	519.12	sentiment	23.055	**662.020**
positive	4.54	34.57	positive	**37.446**	**91.912**
negative	**210.53**	**1307.15**	negative	112.671	1114.727
VADER			VADER		
compound	**48.16**	417.58	compound	26.481	**873.638**
positive	38.7	**103.45**	positive	**58.589**	97.109
negative	**200.26**	1272.16	negative	100.168	**1431.693**
neutral	**220.92**	**1055.16**	neutral	169.21	776.98
EMOJI			EMOJI		
sentiments	13.03	124.46	sentiments	**19.655**	**310.153**
positive	16.74	69.48	positive	11.764	**141.074**
negative	6.54	137.36	negative	**16.035**	**269.929**
neutral	10.17	56.37	neutral	**19.807**	**98.916**

Larger overall *F*-values were observed in Other topic tweets in emojis for all dimensions in circaseptan (except for the positive dimension) and circadian patterns. Additionally, larger overall *F*-values were observed, on the one hand, in circadian patterns for Self-referencing (Other topic) tweets for the following dimensions: LIWC-affect, Hu & Liu-negative, VADER-positive and neutral (LIWC-positive and negative, Hu & Liu-positive and sentiment, VADER-negative and compound); and on the other, for circaseptan patterns for Self-referencing (Other topic) tweets in the following dimensions: LIWC-negative, Hu & Liu-negative and sentiment, VADER-negative, neutral and compound (LIWC-positive and affect, Hu & Liu-positive, VADER-positive).

**Figure 2.**
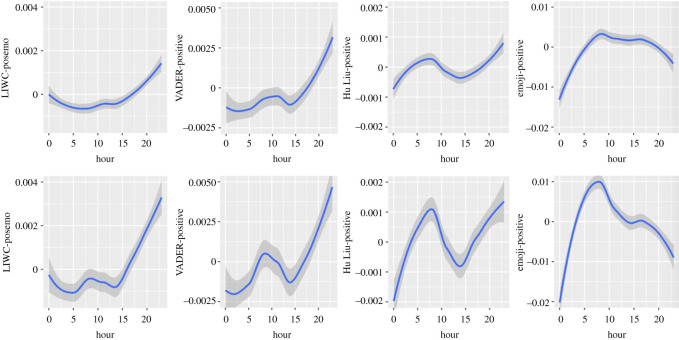
Circadian patterns in positive emotional dimensions (upper half: in Self-referring tweets; lower half: in Other topic tweets).

**Figure 3.**
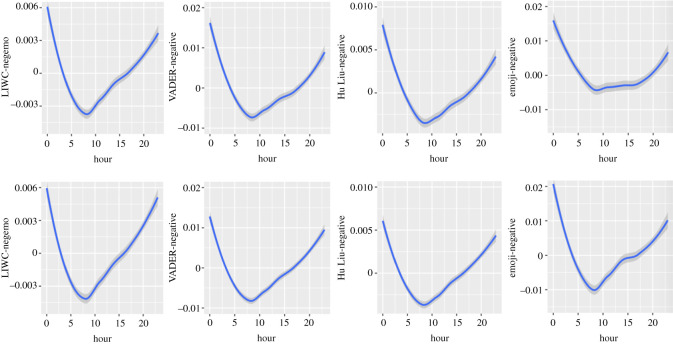
Circadian patterns in negative emotional dimensions (upper half: in Self-referring tweets; lower half: in Other topic tweets).

**Figure 4.**
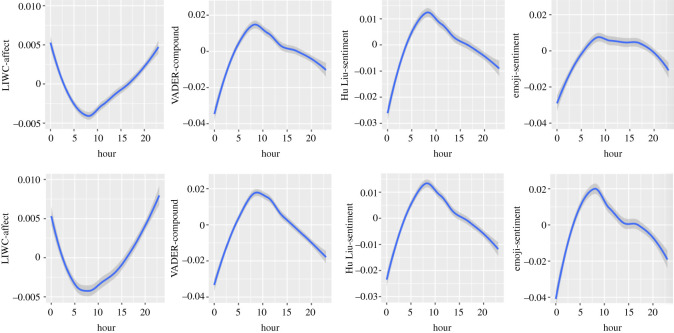
Circadian patterns in composite emotional dimensions (upper half: in Self-referring tweets; lower half: in Other topic tweets).

**Figure 5.**
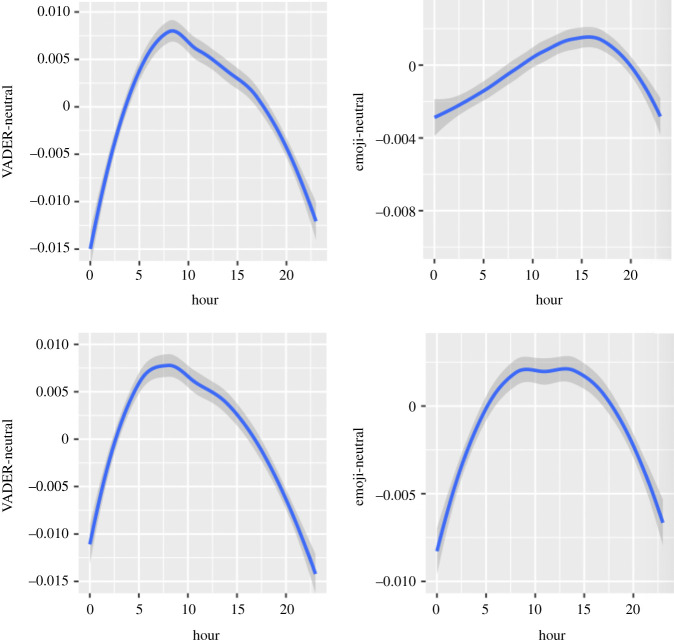
Circadian patterns in neutral emotional dimensions (upper half: in Self-referring tweets; lower half: in Other topic tweets).

**Figure 6.**
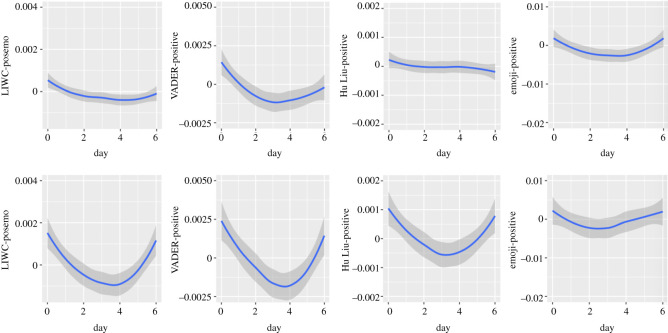
Circaseptan patterns in positive emotional dimensions (upper half: in Self-referring tweets; lower half: in Other topic tweets).

**Figure 7.**
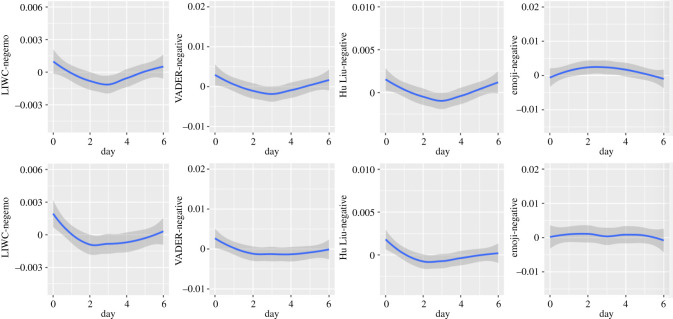
Circaseptan patterns in negative emotional dimensions (upper half: in Self-referring tweets; lower half: in Other topic tweets).

**Figure 8.**
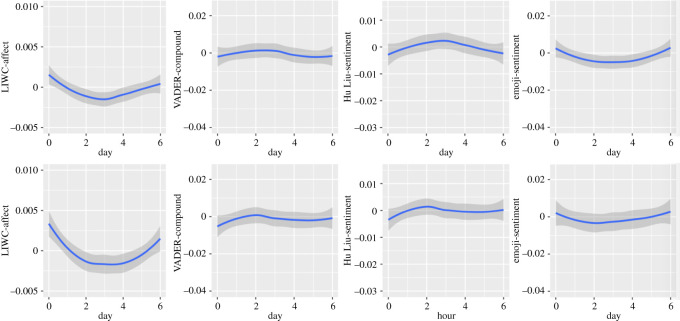
Circaseptan patterns in composite emotional dimensions (upper half: in Self-referring tweets; lower half: in Other topic tweets).

**Figure 9.**
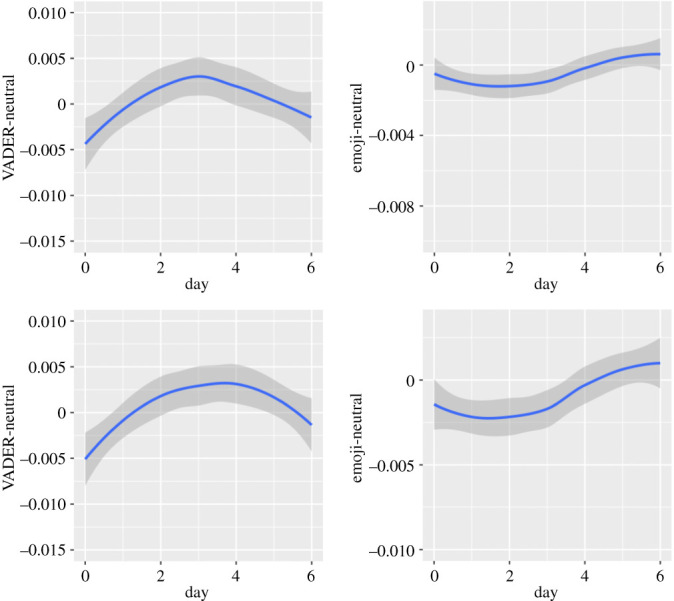
Circaseptan patterns in neutral emotional dimensions (upper half: in Self-referring tweets; lower half: in Other topic tweets).

We now continue with the investigation of the similarities and differences in the patterns of change between Self-referencing tweets and Other topic tweets in the textual analysis and the analysis of emojis. We examine this here visually relying upon figures 2–9, and provide a summary of statistical analyses (mixed-model regression coefficients and *F*-values for each individual polynomial of hour and day). It is worth noting that *p*-values < 0.0001 are not reported in the text for the sake of brevity. These are only significant after the Bonferroni adjustment here (i.e. if a *p*-value is reported, the corresponding trend is non-significant after such adjustment). Comments on the comparative magnitude of the *F*-values relating to the different polynomials are provided in the summary sections.

### 3.1. Circadian patterns

#### 3.1.1. Positive Emotions

##### 3.1.1.1. Linguistic Inquiry and Word Count

In both datasets, positive emotions are highest around 23.00, diminish from midnight to 5.00, and slightly increase afterwards. They remain stable between 11.00 and 13.00 in Self-referencing tweets, whereas a small decrease from 8.00 to 14.00 can be observed in Other topic tweets. Positive emotions then increase drastically from 14.00 to 23.00 (in Self-referencing tweets: Linear trend of Hour: *B* = 1.19*, F*_1, 7 502 937_ = 310.87; Quadratic trend: *B* = 0.73*, F*_1, 7 400 771_ = 116.49; Cubic trend: *B* = −0.03*, F*_1, 7 573 911_ = 0.23, *p* = 0.6296; Quartic trend: *B* = 0.18*, F*_1, 7 553 891_ = 7.49, *p* = 0.0062; Quintic trend: *B* = −0.68*, F*_1, 7 571 210_ =105.0546; in Other topic tweets: Linear trend of Hour: *B* = 2.523*, F*_1, 7 345 330_ = 198.56; Quadratic trend: *B* = 2.666*, F*_1, 7 313 490_ = 222.91; Cubic trend: *B* = 0.351*, F*_1, 7 563 312_ = 4.05, *p* = 0.0440; Quartic trend: *B* = −0.26*, F*_1, 7 525 622_ = 2.15, *p* = 0.1424; Quintic trend: *B* = −2.129*, F*_1, 7 569 779_ = 148.83). This pattern is more marked (i.e. has a larger amplitude) in Other topic tweets than Self-referencing tweets.

#### 3.1.1.2. Valence Aware Dictionary and sEntiment Reasoner

Values in positive emotions are lowest around midnight and tend to increase first slowly then more sharply until 11.00 for Self-referencing tweets (Linear trend of Hour: *B* = 2.36*, F*_1, 7 524 603_ = 242.56; Quadratic trend: *B* = 1.13*, F*_1, 7 437 860_ = 54.00; Cubic trend: *B* = 0.37*, F*_1, 7 577 069_ = 6.12, *p* = 0.0134; Quartic trend: *B* = 0.64*, F*_1, 7 564 702_ = 18.17; Quintic trend: *B* = −2.13*, F*_1, 7 575 904_ = 199.86) and until 9.00 for Other topic tweets (Linear trend of Hour: *B* = 3.409*, F*_1, 7 398 258_ = 112.78; Quadratic trend: *B* = 2.508*, F*_1, 7 367 049_ = 61.39; Cubic trend: *B* = 2.112*, F*_1, 7 571 736_ = 45.69; Quartic trend: *B* = 1.004*, F*_1, 7 545 520_ 10.99, *p* = 0.0009; Quintic trend: *B* = −4.96*, F*_1, 7 575 571_ = 251.58). This is followed by a decline until 14.00 and then a steep increase until 23.00. This pattern is more marked in Other topic tweets than Self-referencing tweets.

#### 3.1.1.3. Hu and Liu

Using the Hu Liu lexicon, the lowest values in positive emotions are observed at midnight. The positive dimension tends to then increase until 9.00 in both Self-referencing and Other topic tweets. This is followed by a steep decline until 14.00. The positive dimension then increases again sharply until 23.00 (in Self-referencing topic tweets: Linear trend of Hour: *B* = 0.36*, F*_1, 7 445 401_ = 33.19; Quadratic trend: *B* = 0.03*, F*_1, 7 299 360_ = 0.31, *p* = 0.5756; Cubic trend: *B* = 0.50*, F*_1, 7 561 718_ = 63.14; Quartic trend: *B* =−0.12*, F*_1, 7 524 252_ = 3.82, *p* = 0.0504; Quintic trend: *B* = −0.54*, F*_1, 7 555 583_ = 72.84; in Other topic tweets: Linear trend of Hour: *B* = −0.51*, F*_1, 7 263 022_ = 10.01, *p* = 0.0016; Quadratic trend: *B* = 0.46*, F*_1, 7 229 848_ = 8.19, *p* = 0.0042; Cubic trend: *B* = 1.97*, F*_1, 7 549 031_ = 155.37; Quartic trend: *B* = −1.364*, F*_1, 7 494 592_ = 73.38; Quintic trend: *B* = −2.277*, F*_1, 7 558 806_ = 207.28. This pattern is more marked in Other topic tweets than Self-referencing tweets.

#### 3.1.1.4. Emoji

Circadian patterns in the positive dimension showed lowest values at midnight, followed by a sharp increase until 8.00. In Self-referencing tweets (Linear trend of Hour: *B* = 1.26*, F*_1, 1 160 346_ = 68.84; Quadratic trend: *B* = −2.23*, F*_1, 1 166 828_ = 215.59; Cubic trend: *B* = −0.18*, F*_1, 1 148 149_ = 1.58, *p* = 0.2089; Quartic trend: *B* = 0.21*, F*_1, 1 152 845_ = 2.16, *p* = 0.1412; Quintic trend: *B* = −1.22*, F*_1, 1 146 950_ = 66.82, a small decrease can then be observed until 17.00, whereas this decrease is sharper in Other topic tweets (Linear trend of Hour*: B* = 2.92, *F*_1, 1 173 894_ = 96.30; Quadratic trend: *B* = −5.90, *F*_1, 1 176 899_ = 398.58; Cubic trend: *B* = 0.97, *F*_1, 1 157 716_ = 11.14, *p* = 0.0008; Quartic trend: *B* = −0.75, *F*_1, 1 163 191_ = 6.52, *p* = 0.0107; Quintic trend: *B* = −4.22, *F*_1, 1 156 577_ = 212.83). After this point, a small increase in the positive dimension is observed in both datasets, followed by a sharp decline. Changes in Other topic tweets are more important than in Self-referencing tweets.

### 3.1.2. Negative Emotions

#### 3.1.2.1. Linguistic Inquiry and Word Count

Negative emotions are highest at midnight in both datasets. They decrease sharply from midnight until early morning and then rise through the day until midnight (in Self-referencing tweets: Linear trend of Hour: *B* = 1.86*, F*_1, 7 503 854_ = 810.91; Quadratic trend: *B* = 4.73*, F*_1, 7 402 034_ = 5212.31; Cubic trend: *B* = −1.85*, F*_1, 7 574 145_ = 823.50; Quartic trend: *B* = 0.47*, F*_1, 7 554 460_ = 52.83; Quintic trend: *B* = 1.21*, F*_1, 7 571 521_ = 351.11; in Other topic tweets: Linear trend of Hour: *B* = 2.67*, F*_1, 7 008 519_ = 470.08; Quadratic trend: *B* = 9.76*, F*_1, 6 987 022_ = 6331.96; Cubic trend: *B* = −3.26*, F*_1, 7 484 990_ = 731.72; Quartic trend: *B* = −0.73*, F*_1, 7 378 469_ = 36.72; Quintic trend: *B* = 2.42*, F*_1, 7 502 240_ = 403.15).

#### 3.1.2.2. Valence Aware Dictionary and sEntiment Reasoner

The negative dimension presents similar patterns to those observed in the LIWC for both Self-referencing tweets (Linear trend of Hour: *B* = 1.50*, F*_1, 7 547 522_ = 102.69; Quadratic trend: *B* = 11.9*, F*_1, 7 483 248_ = 5511.94; Cubic trend: *B* = −2.59*, F*_1, 7 577 249_ = 311.42; Quartic trend: *B* = 0.28*, F*_1, 7 573 443_ = 3.62, *p* = 0.0571; Quintic trend: *B* = 3.29*, F*_1, 7 577 601_ = 501.23) and Other topic tweets (Linear trend of Hour: *B* = 2.70*, F*_1, 7 288 478_ = 120.10; Quadratic trend: *B* = 19.16*, F*_1, 7 259 485_ = 6079.51; Cubic trend: *B* = −5.49*, F*_1, 7 549 782_ = 520.49; Quartic trend: *B* = 0.538*, F*_1, 7 499 452_ = 4.93, *p* = 0.0264; Quintic trend: *B* = 5.353*, F*_1, 7 558 821_ = 495.48). It will, therefore, not be commented upon.

#### 3.1.2.3. Hu and Liu

The Hu & Liu-negative dimension follows a similar pattern to those of the LIWC and VADER (in Self-referencing tweets: Linear trend of Hour: *B* = 0.73*, F*_1, 7 507 514_ = 88.15; Quadratic trend: *B* = 5.89*, F*_1, 7 407 195_ = 5683.43; Cubic trend: *B* = −1.35*, F*_1, 7 575 001_ = 307.27; Quartic trend: *B* = −0.15*, F*_1, 7 556 667_ = 3.85, *p* = 0.0497; Quintic trend: *B* = 1.75*, F*_1, 7 572 680_ = 518.72; in Other topic tweets: Linear trend of Hour: *B* = 1.15*, F*_1, 7 044 803_ = 67.28; Quadratic trend: *B* = 9.55*, F*_1, 7 022 530_ = 4705.75; Cubic trend: *B* = −2.77*, F*_1, 7 493 329_ = 409.89; Quartic trend: *B* = −0.105*, F*_1, 7 394 020_ = 0.58, *p* = 0.4447; Quintic trend: *B* = 2.85*, F*_1, 7 509 638_ = 436.49.

#### 3.1.2.4. Emoji

Again, the negative dimension shows strong similarities with the other tools, when it comes to circadian patterns, with a less steady increase between 10.00 and 15.00 in Self-referencing tweets (Linear trend: *B* = −1.37*, F*_1, 1 162 334_ = 73.43; Quadratic trend: *B* = 3.66*, F*_1, 1 168 259_ = 518.97; Cubic trend: *B* = −0.17*, F*_1, 1 151 160_ = 1.12, *p* = 0.2889; Quartic trend: *B* = –0.64*, F*_1, 1 155 465_ = 16.24, *p* < 0.0001; Quintic trend: *B* = 1.50*, F*_1, 1 150 063_ = 89.29, *p* < 0.0001; in Other topic tweets: Linear trend: *B* = −1.04, *F*_1, 1 182 465_ = 20.24; Quadratic trend: *B* = 7.25, *F*_1, 1 182 227_ = 992.77; Cubic trend: *B* = −2.01, *F*_1, 1 176 662_ = 78.62; Quartic trend: *B* = 0.19, *F*_1, 1 179 728_ = 0.73, *p* = 0.3932; Quintic trend: *B* = 3.74, *F*_1, 1 176 301_ = 273.52). The global pattern is more marked in Other topic tweets than Self-referencing tweets.

### 3.1.3. Composite Dimensions

#### 3.1.3.1. Linguistic Inquiry and Word Count

A similar pattern to negative emotions can be observed for the LIWC-affect dimension (in Self-referencing tweets: Linear trend of Hour: *B* = 3.11*, F*_1, 7 500 552_ = 1289.78; Quadratic trend: *B* = 5.01*, F*_1, 7 397 230_ = 3305.96; Cubic trend: *B* = −1.81*, F*_1, 7 573 336_ = 444.96; Quartic trend: *B* = 0.57*, F*_1, 7 552 483_ = 45.06; Quintic trend: *B* = 0.41*, F*_1, 7 570 451_ = 22.86; in Other topic tweets: Linear trend of Hour: *B* = 4.66*, F*_1, 7 288 021_ = 500.67; Quadratic trend: *B* = 11.40*, F*_1, 7 256 587_ = 3017.10; Cubic trend: *B* = −2.42*, F*_1, 7 552 076_ = 141.43; Quartic trend: *B* = −0.27*, F*_1, 7 502 287_ = 1.74, *p* = 0.1869; Quintic trend: *B* = 0.07*, F*_1, 7 560 991_ = 0.12, *p* = 0.7215).

#### 3.1.3.2. Valence Aware Dictionary and sEntiment Reasoner

The compound dimension follows the inverse pattern of changes to its negative dimension: lowest at midnight, increasing until 8.00, and then decreasing (in Self-referencing tweets: Linear trend of Hour: *B* = 0.53*, F*_1, 7 570 780_ = 1.15, *p* = 0.2829; Quadratic trend: *B* = −0.19*, F*_1,7 535 316_ = 1402.58; Cubic trend: *B* = 7.17*, F*_1, 7 568 715_ = 214.20; Quartic trend: *B* = 1.15*, F*_1, 7 577 276_ = 5.49, *p* = 0.0191; Quintic trend: *B* = −11.1*, F*_1, 7 571 044_ = 512.10; in Other topic tweets: Linear trend of Hour: *B* = −0.73*, F*_1, 7 488 173_ = 1.10, *p* = 0.2927; Quadratic trend: *B* = −39.95*, F*_1, 7 462 899_ = 3356.40; Cubic trend: *B* = 12.95*, F*_1, 7 577 590_ = 371.70; Quartic trend: *B* = 8.47*, F*_1, 7 570 971_ = 151.93; Quintic trend: *B* = −1.62*, F*_1, 7 576 317_ = 583.27).

#### 3.1.3.3. Hu and Liu

The Hu Liu sentiment dimension shows strong similarities with the VADER-compound dimension in both datasets: (in Self-referencing tweets: Linear trend of Hour: *B* = −1.83*, F*_1, 7 518 334_ = 23.76; Quadratic trend: *B* = −0.17*, F*_1, 7 425 083_ = 1920.73: Cubic trend: *B* = 6.63, *F*_1, 7 576 641_ = 315.67; Quartic trend: *B* = 0.37*, F*_1, 7 562 233_ = 0.097, *p* = 0.3252; Quintic trend: *B* = −7.21*, F*_1, 7 575 125_ = 373.83; in Other topic tweets: Linear trend of Hour: *B* = −1.95*, F*_1, 7 328 333_ = 11.61, *p* = 0.0007; Quadratic trend: *B* = −27.75*, F*_1, 7 296 595_ = 2363.45; Cubic trend: *B* = 11.86*, F*_1, 7 560 195_ = 452.00; Quartic trend: *B* = 0.51*, F*_1, 7 518 851_ = 0.85, *p* = 0.3557; Quintic trend: *B* = −13. 50*, F*_1, 7 567 445_ = 546.74).

#### 3.1.3.4. Emoji

The emoji sentiment dimension follows a pattern close to identical to its positive dimension (in Self-referencing tweets: Linear trend of Hour: *B* = 2.63*, F*_1, 1 158 923_ = 89.96; Quadratic trend: *B* = −5.83*, F*_1, 1 165 328_ = 435.06; Cubic trend: *B* = −0.37*, F*_1, 1 147 056_ = 1.81, *p* = 0.1775; Quartic trend: *B* = −0.87*, F*_1, 1 151 592_ = 10.51, *p* = 0.0012; Quintic trend: *B* = −2.72*, F*_1, 1 145 813_ = 97.52; in Other topics tweets: Linear trend of Hour: *B* = 4.02, *F*_1, 1 171 778_ = 105.89; Quadratic trend: *B* = −12.33, *F*_1, 1 175 134_ = 1014.50; Cubic trend: *B* = 2.65, *F*_1, 1 155 302_ = 48.63; Quartic trend: *B* = −0.60, *F*_1, 1 160 704_ = 2.45, *p* = 0.1173; Quintic trend: *B* = −7.74, *F*_1, 1 154 089_ = 416.61). Again, the pattern of change is more important in Other topic tweets than Self-referencing tweets.

### 3.1.4. Neutral Dimensions

#### 3.1.4.1. Valence Aware Dictionary and sEntiment Reasoner

The neutral dimension follows a similar circadian pattern to the compound dimension with a steeper decline in both datasets, but more marked in Other topic tweets (in Self-referencing tweets: Linear trend of Hour: *B* = −3.88*, F*_1, 7 531 127_ = 457.18; Quadratic trend: *B* = −12.39*, F*_1, 7 451 705_ = 4598.67; Cubic trend: *B* = 2.27*, F*_1, 7 577 382_ = 159.35; Quartic trend: *B* = −1.0*, F*_1, 7 567 100_ = 30.51; Quintic trend: *B* = −1.20*, F*_1, 7 576 558_ = 44.93; in Other topic tweets: Linear trend of Hour: *B* = −6.05*, F*_1, 7 349 973_ = 279.66; Quadratic trend: *B* = −21.24*, F*_1, 7 318 679_ = 3466.70; Cubic trend: *B* = 3.24*, F*_1, 7 563 762_ = 84.32; Quartic trend: *B* = −1.45*, F*_1, 7 526 859_ = 16.72; Quintic trend: *B* = −0.29*, F*_1, 7 570 095_ = 0.67, *p* = 0.4116).

#### 3.1.4.2. Emoji

Circadian patterns in the neutral dimension have their lowest values around midnight. In Self-referencing tweets, this is followed by a marked increase until 3.00, itself followed by a sharp decline until midnight (Linear trend of Hour: *B* = 0.53*, F*_1, 1 157 024_ = 44.10; Quadratic trend: *B* = −1.03*, F*_1, 1 164 249_ = 161.82; Cubic trend: *B* = −0.63*, F*_1, 1 143 542_ = 61.74; Quartic trend: *B* = 0.32*, F*_1, 1 148 713_ = 16.39; Quintic trend: *B* = −0.007*, F*_1, 1 142 184_ = 0.008, *p* = 0.9272; whereas Other topic tweets feature a sharper increase from midnight to 8.00, followed by a period of stabilization until 13.00 and a sharp decline until midnight (Linear trend of Hour: *B* = 1.49, *F*_1,1 178 139_ = 70.19; Quadratic trend: *B* = −3.50, *F*_1,1 180 199_ = 396.41; Cubic trend: *B* =−0.80, *F*_1,1 163 498_ = 21.63; Quartic trend: *B* = 0.61, *F*_1,1 168 883_ = 12.17, *p* = 0.0005; Quintic trend: *B* = −0.37, *F*_1,1 162 559_ = 4.70, *p* = 0.0302). The amplitude of the pattern is larger in Other topic tweets than Self-referencing tweets.

#### 3.1.5. Summary

We have found strong similarities in the negative emotion dimensions between (i) Self-referencing and Other topic tweets, (ii) textual analyses with all instruments, and (iii) emojis coded for emotional expressiveness and textual analyses with all instruments. The LIWC and VADER exhibited similar patterns in both datasets for positive emotions, whereas a distinct N-shaped pattern was observed in the Hu & Liu lexicon in Self-referencing tweets and Other topic tweets (more marked). The coding of emojis for positive emotions displayed a distinct pattern from all textual analysis instruments. Considering the composite measures, the circadian pattern displayed by the emoji sentiment measure was similar to the VADER and Hu & Liu coding (in both datasets; reversed U-shaped, attenuated in Self-referencing tweets), whereas the LIWC-Affect dimension exhibited the opposite pattern (U-shaped). The coding of neutral emotional content was similar across datasets (reversed U-shaped) and in both tools. Finally, for most of the comparisons between datasets, the observed pattern was more marked in Other topic tweets than Self-referencing tweets, which is not always consistent with the *F*-values presented in table 2.

## 3.2. Circaseptan patterns

### 3.2.1. Positive Emotions

#### 3.2.1.1. Linguistic Inquiry and Word Count

Positive emotions are highest on Sundays. There is a decline in positive emotions throughout the week until Friday and an increase on both Saturday and Sunday. This increase is less marked for Self-referencing tweets compared with Other topic tweets (in Self-referencing tweets: Linear trend of Day: *B* = −0.5*, F*_1, 7 576 484_ = 61.34; Quadratic trend: *B* = 0.4*, F*_1, 7 572 765_ = 36.04; Cubic trend: *B* = 0.15*, F*_1, 7 572 910_ = 5.27, *p* = 0.0217; Quartic trend: *B* = 0.1*, F*_1, 7 569 522_ = 2.44, *p* = 0.1184; Quintic trend: *B* = −0.009*, F*_1, 7 565 323_ = 0.02, *p* = 0.9865; in Other topic tweets: Linear trend of Day: *B* = −0.29*, F*_1, 7 564 858_ = 2.78, *p* = 0.0952; Quadratic trend: *B* = 3.042*, F*_1, 7 577 506_ = 310.50; Cubic trend: *B* = 0.60*, F*_1, 7 553 293_ = 12.35, *p* = 0.0004; Quartic trend: *B* = 0.21*, F*_1, 7 547 527_ = 1.49, *p* = 0.2216; Quintic trend: *B* = −0.23*, F*_1, 7 538 334_ = 1.86, *p* = 0.1722).

#### 3.2.1.2. Valence Aware Dictionary and sEntiment Reasoner

Circaseptan patterns in the VADER-positive dimension are similar to the LIWC, but with a lesser difference between Other topic tweets and Self-referencing tweets in terms of amplitude (in Self-referencing tweets: Linear trend of Day: *B* = −1.41*, F*_1, 7 573 072_ = 89.15; Quadratic trend: *B* = 1.47*, F*_1, 7 576 447_ = 95.09; Cubic trend: *B* = 0.42*, F*_1, 7 567 351_ = 7.96, *p* = 0.0048; Quartic trend: *B* = −0.14*, F*_1, 7 562 614_ = 0.93, *p* = 0.3352; Quintic trend: *B* = 0.10*, F*_1, 7 557 255_ = 0.43, *p* = 0.5092; in other topic tweets: Linear trend of Day: *B* = −1.281*, F*_1, 7 556 106_ = 17.28; Quadratic trend: *B* = 4.862*, F*_1, 7 576 370_ = 247.40; Cubic trend: *B* = 1.462*, F*_1, 7 542 387_ = 22.62; Quartic trend: *B* = −0.28*, F*_1, 7 535 877_ = 0.82, *p* = 0.3650; Quintic trend: *B* = −0.65*, F*_1, 7 525 575_ = 4.55, *p* = 0.0327).

#### 3.2.1.3. Hu and Liu

The pattern of circaseptan change in the positive dimension differs in Self-referencing tweets (highest value on Sunday, followed by a gradual decline through the week until the lowest value is reached on Saturday; Linear trend of Day: *B* = −0.28*, F*_1, 7 577 351_ = 19.25; Quadratic trend: *B* = −0.08*, F*_1, 7 560 333_ = 1.77, *p* = 0.1825; Cubic trend: *B* = −0.03*, F*_1, 7 577 112_ = 0.23, *p* = 0.6273; Quartic trend: *B* = −0.08*, F*_1, 7 575 520_ = 1.42, *p* = 0.233; Quintic trend: *B* = 0.006*, F*_1, 7 572 779_ = 0.01, *p* = 0.9234) and Other topic tweets (highest value on Sunday followed by a steep decline until Thursday, then a steep increase until Saturday; Linear trend of Day: *B* = −0.41*, F*_1, 7 571 372_ = 7.00, *p* = 0.0081; Quadratic trend: *B* = 2.017*, F*_1, 7 575 697_ = 166.15; Cubic trend: *B* = 0.346*, F*_1, 7 561 744_ = 4.93, *p* = 0.0263; Quartic trend: *B* = −0.297*, F*_1, 7 556 546_ = 3.627, *p* = 0.057; Quintic trend: *B* = −0.355*, F*_1, 7 548 092_ = 5.21, *p* = 0.0224.

#### 3.2.1.4. Emoji

Circaseptan patterns in the positive dimension are similar to those observed in the LIWC and VADER lexicon, with stronger increases from Wednesday to the end of the week (in Self-referencing tweets: Linear trend of Day: *B* = −0.32*, F*_1, 1 142 618_ = 4.47, *p* = 0.0344; Quadratic trend: *B* = 1.17*, F*_1, 1 148 717_ = 61.72; Cubic trend: *B* = 0.31*, F*_1, 1 138 873_ = 4.27, *p* = 0.0387; Quartic trend: *B* = 0.54*, F*_1, 1 137 678_ = 13.12, *p* = 0.0003; Quintic trend: *B* = 0.06*, F*_1, 1 134 525_ = 0.15, *p* = 0.7008; in Other topic tweets: *B* = 0.08, *F*_1,1 151 888_ = 0.07, *p* = 0.7916; Quadratic trend: *B* = 2.06, *F*_1,1 160 527_ = 46.74; Cubic trend: *B* = −0.57, *F*_1,1 149 265_ = 3.86, *p* = 0.0495; Quartic trend: *B* = 0.12, *F*_1,1 146 131_ = 0.18, *p* = 0.6745; Quintic trend: *B* = 0.80, *F*_1,1 142 342_ = 7.95, *p* = 0.0048).

### 3.2.2. Negative Emotions

#### 3.2.2.1. Linguistic Inquiry and Word Count

Negative emotions are highest on Sundays, declining from Mondays to Wednesdays and rising again afterwards, more markedly in Self-referencing tweets (in Self-referencing tweets: Linear trend of Day: *B* = 1.44*, F*_1, 7 576 347_ = 5.00, *p* = 0.0254; Quadratic trend: *B* = 1.74*, F*_1, 7 573 014_ = 726.77; Cubic trend: *B* = −0.93*, F*_1, 7 572 629_ = 210.11; Quartic trend: *B* = −0.87*, F*_1, 7 569 146_ = 183.61; Quintic trend: *B* = −0.61*, F*_1, 7 564 857_ = 90.36; in Other topic tweets: Linear trend of Day: *B* = −1.43*, F*_1, 7 576 720_ = 143.00; Quadratic trend: *B* = 3.32*, F*_1, 7 550 466_ = 773.57; Cubic trend: *B* = −1.00*, F*_1, 7 576 787_ = 70.82; Quartic trend: *B* = −0.59*, F*_1, 7 575 053_ = 24.17; Quintic trend: *B* = −0.05*, F*_1, 7 571 145_ = 0.19, *p* = 0.6605).

#### 3.2.2.2. Valence Aware Dictionary and sEntiment Reasoner

The negative dimension presents similar patterns to those observed in the LIWC and Hu Liu lexicon for both Self-referencing tweets (Linear trend of Day: *B* = −0.02*, F*_1, 7 567 271_ = 0.01, *p* = 0.9139; Quadratic trend: *B* = 3.67*, F*_1, 7 577 565_ = 626.30. Cubic trend: *B* = −1.90*, F*_1, 7 559 874_ = 170.35; Quartic trend: *B* = −1.74*, F*_1, 7 554 132_ = 141.86; Quintic trend: *B* = −1.15*, F*(1, 7548067) = 61.93) and Other topic tweets (Linear trend of Day: *B* = −2.40*, F*_1, 7 573 177_ = 102.27; Quadratic trend: *B* = 4.52*, F*_1, 7 575 225_ = 361.24; Cubic trend: *B* = −1.12*, F*_1, 7 565 299_ = 22.15; Quartic trend: *B* = 0.85*, F*_1, 7 560 947_ = 12.86, *p* = 0.0003; Quintic trend: *B* = −0.32, *p* = 0.1735*, F*_1, 7 553 824_ = 1.85, *p* = 0.1735). Hence, we will not comment further on this dimension.

#### 3.2.2.3. Hu and Liu

The negative dimension follows similar patterns to that of the LIWC and VADER instruments (in Self-referencing tweets: Linear trend of Day: *B* = 0.14*, F*_1, 7 575 729_ = 3.54, *p* = 0.06; Quadratic trend: *B* = 2.03*, F*_1, 7 573 938_ = 696.15; Cubic trend: *B* = −0.82*, F*_1, 7 571 446_ = 116.40); Quartic trend: *B* = −0.93*, F*_1, 7 567 588_ = 148.04; Quintic trend: *B* = −0.71*, F*_1, 7 562 946_ = 87.69; in Other topic tweets: Linear trend of Day: *B* = −0.89*, F*_1, 7 577 204_ = 43.04; Quadratic trend: *B* = 2.76*, F*_1, 7 553 978_ = 416.56; Cubic trend: *B* = −1.34*, F*_1, 7 576 532_ = 98.84; Quartic trend: *B* = −0.15*, F*_1, 7 574 587_ = 1.24, *p* = 0.2652; Quintic trend: *B* = −0.23*, F*_1, 7 570 467_ = 2.75, *p* = 0.0974.

#### 3.2.2.4. Emoji

Circaseptan patterns of Self-referencing tweets in the negative dimension appear to be inverted for emojis compared with the textual analysis tools: the negative dimension was highest during the workweek with a peak on Tuesday. The peak was preceded by an increase from Sunday and followed by a decrease until Saturday in both datasets (Self-referencing tweets: *B* = 0.06*, F*_1, 1 146 083_ = 0.13, *p* = 0.7157; Quadratic trend: *B* = −0.71*, F*_1, 1 151 679_ = 20.21; Cubic trend: *B* = −0.95*, F*_1, 1 142 645_ = 0.36, *p* = 0.5485; Quartic trend: *B* = −0.45*, F*_1, 1 141 548_ = 8.19, *p* = 0.0042; Quintic trend: *B* = −0.31*, F*_1, 1 138 651_ = 3.82, *p* = 0.0505). In Other topics tweets, the pattern was interrupted by a decrease on Wednesday (Linear trend of Day: *B* = 0.09, *F*_1, 1 173 055_ = 0.13, *p* = 0.7168; Quadratic trend: *B* = −0.98, *F*_1, 1 177 910_ = 17.34; Cubic trend: *B* = −0.47, *F*_1, 1 171 383_ = 4.24, *p* = 0.0395; Quartic trend: *B* = −1.35, *F*_1, 1 169 237_ = 36.17; Quintic trend: *B* = −0.98, *F*_1, 1 166 839_ = 19.58).

### 3.2.3. Composite Dimensions

#### 3.2.3.1. Linguistic Inquiry and Word Count

Again, the pattern for LIWC-Affect dimension is very similar to that observed for Negative emotions (in Self-referencing tweets: Linear trend of Day: *B* = −0.32*, F*_1, 7 576 763_ = 13.93, *p* = 0.0002; Quadratic trend: *B* = 2.21*, F*_1, 7 572 159_ = 669.41; Cubic trend: *B* = −0.85*, F*_1, 7 573 500_ = 99.28; Quartic trend: *B* = −0.79*, F*_1, 7 570 314_ = 87.34; Quintic trend: *B* = −0.61*, F*_1, 7 566 304_ = 50.62; in Other topic tweets: Linear trend of Day: *B* = −1.52*, F*_1, 7 571 087_ = 57.56; Quadratic trend: *B* = 6.24*, F*_1, 7 576 021_ = 965.02; Cubic trend: *B* = −0.37*, F*_1, 7 561 783_ = 3.35, *p* = 0.0672; Quartic trend: *B* = 0.68*, F*_1, 7 556 809_ = 11.57, *p* = 0.0007; Quintic trend: *B* = −0.30*, F*_1, 7 548 742_ = 2.24, *p* = 0.1346).

#### 3.2.3.2. Valence Aware Dictionary and sEntiment Reasoner

In Self-referencing tweets (Linear trend of Day: *B* = −2.83*, F*_1, 7 549 359_ = 33.91; Quadratic trend: *B* = −2.69*, F*_1, 7 571 262_ = 30.21; Cubic trend: *B* = 6.20*, F*_1, 7 538 794_ = 162.50; Quartic trend: *B* = 1.66*, F*_1, 7 530 944_ = 11.71, *p* = 0.0006; Quintic trend: *B* = 0.82*, F*_1, 7 523 328_ = 2.87, *p* = 0.09), the lowest values in the VADER-compound dimension are observed on Sunday and Friday. An increase can be observed from Sunday to Tuesday, followed by a decrease from Wednesday until Friday followed by a small increase. For Other topic tweets (Linear trend of Day: *B* = 1.39*, F*_1, 7 535 372_ = 4.40, *p* = 0.0358; Quadratic trend: *B* = −4.70*, F*_1, 7 566 486_ = 50.01; Cubic trend: *B* = 5.39*, F*_1, 7 519 374_ = 64.38; Quartic trend: *B* = −2.27*, F*_1, 7 512 252_ = 11.76, *p* = 0.0006; Quintic trend: *B* = −0.95*, F*_1, 7 500 957_ = 2.06, *p* = 0.1516), the pattern is similar with the exceptions that the lowest value was observed on Sunday only and that declines in VADER compound were lower during the workweek compared to Self-referencing tweets.

#### 3.2.3.3. Hu and Liu

The Hu & Liu sentiment dimension shows the lowest values on Sunday for both Self-referencing tweets and Other topic tweets (in Self-referencing tweets: Linear trend of Day: *B* = −1.32*, F*_1, 7 573 800_ = 12.72, *p* < 0.0004; Quadratic trend: *B* = −5.28*, F*_1, 7 575 859_ = 200.18; Cubic trend: *B* = 3.27*, F*_1, 7 568 287_ = 77.67; Quartic trend: *B* = 1.85*, F*_1, 7 563 638_ = 24.92; Quintic trend: *B* = 1.82*, F*_1, 7 558 301_ = 24.12; in Other topic tweets: Linear trend of Day: *B* = 1.21, *F* = _1, 7 567 247_ = 4.82, *p* = 0.28; Quadratic trend: *B* = −3.68*, F*_1, 7 577 424_ = 44.37; Cubic trend: *B* = 3.86*, F*_1, 7 556 421_ = 49.36; Quartic trend: *B* = −1.69*, F*_1, 7 550 915_ = 9.38, *p* = 0.0022; Quintic trend: *B* = −1.48, *F*_1, 7 542 099_ = 7.32, *p* = 0.0068. Values increase until Wednesday for Self-referencing tweets and until Tuesday for Other topic tweets. This is followed by a decrease until Saturday for Self-referencing tweets, and until Thursday for Other topic tweets (which feature stable values until the weekend). Globally, the pattern we observe is similar to the VADER-Compound dimension.

#### 3.2.3.4. Emoji

The Emoji sentiment dimension is quite different from the Vader-compound and Hu & Liu-sentiment but quite similar to the LIWC-affect dimension: values are higher in the weekend. They decrease until Tuesday and increase again from Wednesday (in Self-referencing tweets: Linear trend of Day: *B* = −0.38*, F*_1, 1 141 752_ = 1.94, *p* = 0.1634; Quadratic trend: *B* = 1.88*, F*_1, 1 147 651_ = 46.33; Cubic trend: *B* = −0.40*, F*_1, 1 138 101_ = 2.16, *p* = 0.1416; Quartic trend: *B* = 0.98*, F*_1, 1 136 949_ = 12.98; Quintic trend: *B* = 0.36*, F*_1, 1 133 882_ = 1.78, *p* = 0.1827; in Other topics tweets: Linear trend of Day: *B* = −0.13, *F*_1, 1 149 528_ = 0.11, *p* = 0.7393; Quadratic trend: *B* = 3.04, *F*_1, 1 158 176_ = 59.59; Cubic trend: *B* = −0.07, *F*_1, 1 146 926_ = 0.04, *p* = 0.8502; Quartic trend: *B* = 1.46, *F*_1, 1 143 844_ = 15.12, *p* = 0.0001; Quintic trend: *B* = 1.78, *F*_1, 1 140 068_ = 23.11).

### 3.2.4. Neutral Dimensions

#### 3.2.4.1. Valence Aware Dictionary and sEntiment Reasoner

The lowest values in the neutral emotional dimension are observed on Sunday. From that day, values increase until Wednesday in Self-referencing tweets (Linear trend of Day: *B* = 1.45*, F*_1, 7 572 370_) = 65.82; Quadratic trend: *B* = −5.15*, F*_1, 7 576 933_ = 821.87; Cubic trend: *B* = 1.48*, F*_1, 7 566 535_ = 69.13; Quartic trend: *B* = 1.88*, F*_1, 7 561 780_ = 111.70; Quintic trend: *B* = 1.05*, F*_1, 7 556 478_ = 34.85) and until Thursday in Other topic tweets (Linear trend of Day: *B* = 3.65*, F*_1, 7 565 140_ = 109.93; Quadratic trend: *B* = −9.38*, F*_1, 7 577 621_ = 723.37; Cubic trend: *B* = −0.39*, F*_1, 7 553 768_ = 1.22, *p* = 0.2688; Quartic trend: *B* = −0.58*, F*_1, 7 548 099_ = 2.77, *p* = 0.0960; Quintic trend: *B* = −0.99*, F*_1, 7 539 059_ = 8.22, *p* = 0.0041). This is followed by a decrease until the end of the week.

#### 3.2.4.2. Emoji

Circaseptan patterns in the neutral dimension are close to S-shaped (inclined to the right) with lowest values on Tuesday for Self-referencing tweets (Linear trend of Day: *B* = 0.45*, F*_1, 1 137 475_ = 34.21, *p* < 0.0001; Quadratic trend: *B* = 0.25*, F*_1, 1 144 189_ = 10.21, *p* = 0.0014; Cubic trend: *B* = −0.20*, F*_1, 1 133 344_ = 6.47, *p* = 0.0110; Quartic trend: *B* = −0.24*, F*_1, 1 132 033_ = 0.09, *p* = 0.7642; Quintic trend: *B* = 0.003*, F*_1, 1 128 562_ = 0.002, *p* = 0.9614 and on Monday and Tuesday for Other topic tweets (Linear trend of Day: *B* = 1.35, *F*_1, 1 157 757_ = 57.18; Quadratic trend: *B* = 0.59, *F*_1, 1 166 053_ = 10.80, *p* = 0.0010; Cubic trend: *B* = −0.70, *F*_1, 1 155 170_ = 15.97; Quartic trend: *B* = −0.32, *F*_1, 1 152 020_ = 3.48, *p* = 0.0621; Quintic trend: *B* = −0.26, *F*_1, 1 148 319_ = 2.44, *p* = 0.1181).The amplitude of the pattern is larger in Other topic tweets than Self-referencing tweets.

#### 3.2.5. Summary

The negative emotion dimension of all textual analysis tools featured similar patterns (U-shaped). In emojis, the pattern of NA appeared to be reverse U-shaped instead (Self-referencing tweets particularly). A U-shaped pattern was observed in positive emotions as well as in emoji coding and for all textual analysis tools in both datasets, but the Hu & Liu in Self-referencing tweets, which featured a gradual decline through the week. The composite dimensions displayed a U-shaped pattern in emojis and for the LIWC, an N-shaped pattern for the VADER in both datasets and the Hu & Liu for Other topic tweets. For this dimension, the Hu Liu featured a reversed U-shaped pattern in Self-referencing tweets. Finally, neutral expression of emotions in emojis followed an S-shaped pattern whereas a reversed U-shaped pattern was found for the textual analysis using the VADER.

### 3.3. Relative contribution of the individual polynomial trends

We provide example plots of linear, quadratic, cubic, quartic and quintic functions before commenting on the relative contribution of the individual polynomial trends to the overall temporal trajectories. In our case, these can be interpreted as the shape that a temporal pattern would follow in the case of it being affected by only a single polynomial trend. For instance, a positive quadratic regression coefficient would result in a U-shaped pattern as it has been observed in all negative emotional dimensions in our study in circadian patterns of Self-referring as well as Other topics tweets).

For each temporal trajectory, the complete patterns depend upon the sign of each polynomial trend (here from linear to quintic) entered as a predictor and its magnitude. Because these predictors are scaled differently, their relative magnitude cannot be known directly from the coefficients. We used the individual *F*-value as an approximation of the magnitude of the different trends. For instance, the largest *F*-values are often those relating to the quadratic trends in both datasets for both circadian and circaseptan patterns. This corresponds to what can be observed, for instance, in figure 3. The trends not mentioned below contribute comparatively marginally to the overall patterns.

#### 3.3.1. Circadian patterns

**Figure 10.**
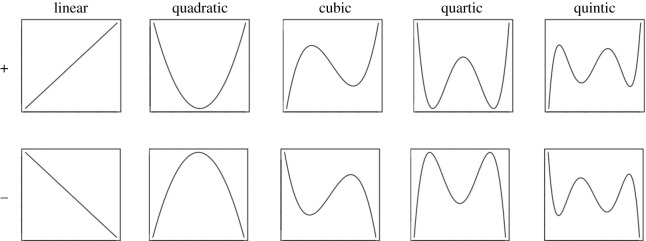
Graphing of different polynomial equations. The temporal trajectory represented by a single positive/negative trend (linear, quadratic, cubic quartic or quantic) in the presented analyses would look similar to the patterns presented on the plots on the top/bottom panel if all other coefficients had a value of 0.

The negative dimensions all feature a large positive quadratic trend in Self-referring and Other topic tweets, based upon the individual *F*-values. Quadratic trends are dominant in both datasets across composite dimensions (LIWC-affect, VADER-compound, Hu Liu-sentiment and emoji-sentiment). The sign for this trend is negative—leading to a trajectory largely similar to the bottom panel of figure 10 under Quadratic, except for LIWC-affect (pattern like Quadratic in figure 10 top panel). More differences can be observed in the positive affective dimensions. The dominant trends are positive linear and positive quadratic in both datasets for the LIWC, positive linear and negative quintic in both datasets for VADER, positive cubic and negative quintic in both datasets for the Hu Lui lexicon, and negative quadratic in the emoji coding for both datasets, with a large magnitude of the quintic trend in Other topic tweets (negative). Finally, the neutral categories (VADER, emoji coding) feature dominant negative quadratic trends in both datasets.

#### 3.3.2. Circaseptan patterns

The negative dimensions feature a dominant positive quadratic trend in both datasets in the textual analysis tools, as well as for the emoji coding in Self-referencing tweets. In Other topic tweets, the dominant trends are quadratic and quartic, both negative. A relatively important quadratic trend is found for all the composite dimensions (negative in LIWC-Affect; positive in VADER-Compound, Hu & Liu-sentiment and Emoji-sentiment), accompanied by large magnitudes in the cubic trend (positive) of the VADER-compound and Hu & Liu-sentiment dimensions in Other topic tweets. The positive dimensions of Other topic tweets feature this dominance of positive quadratic trends. The positive dimensions in Self-referring tweets also feature such dominance of positive quadratic trends for the VADER (accompanied by a strong linear negative trend) and emoji-positive dimensions, whereas the LIWC and Hu & Liu-positive dimensions feature a dominant negative linear trend accompanied by a strong positive quadratic trend in the case of the LIWC. Finally, the neutral dimensions feature dominant positive linear trends in the case of emoji and dominant negative quadratic trends for VADER.

## 4. Discussion

Our study extended previous studies [30,46,47,83] by comparing content coded for expressed emotions (i) between Self-referencing tweets and Other topic tweets, (ii) between different tools for textual analysis, and (iii) between said textual analysis and emoji coding. In sum, our statistical analyses have confirmed the presence of circadian and circaseptan patterns in Self-referencing tweets and in Other topic tweets. Our study also showed that there exist similarities and differences in these patterns when comparing Self-referencing tweets with Other topic tweets, the different textual analysis tools as well as the emoji coding. Conceptual differences and general differences in the performances shown by VADER and LIWC for the analysis of Twitter data were explained in the Method (see [80] for a systematic review).

### 4.1. Similarities and differences in pattern shapes

Circadian patterns in negative emotions were similar in shape across textual analysis instruments, datasets and in the comparison between the emoji coding and textual analysis coding. This occurred in Self-referencing tweets and Other topic tweets. Values sharply decreased from midnight to 8.00 and increased steadily throughout the day until midnight. Sleep may serve to reset negative emotions [30] and is in line with research demonstrating that overnight sleep in general, and REM sleep in particular, modulates affective neural systems [84]. In line with previous studies [30], circadian patterns began increasing during working hours and such a trend continued until the late night. Emoji showed a slight variation to this pattern as negative emotions remained mostly stable from 9.00 to 17.00 in Self-referencing tweets and from 13.00 to 17.00 in Other topic tweets, but overall this corresponded to the textual analyses of circadian patterns in negative emotions. In the literature, these have been used as a baseline for the early detection of depression in individuals, such as morning depression symptoms [85]. We suggest the analysis of emojis could also be indicative.

Negative emotions also featured quite similar circaseptan pattern shapes between datasets and textual analysis tools. In Self-referencing (Other topic), tweets decreased from Sunday to Wednesday (Tuesday) and increased until Saturday. Decreases and increases occurred gradually throughout the week. These results do not correspond to what was observed in previous research using LIWC exclusively [47]. In Wang *et al*. [47], negative emotions decreased gradually from Monday to Thursday, then experienced a marked decrease on Friday before showing a sharp increase during the weekend. Our analysis did not show a sharp Friday dip followed by a marked increase from Friday to Sunday.

Throughout the week, the expression of negative emotions with emoji increased from Sunday to Wednesday in Self-referencing tweets and from Sunday to Tuesday in Other topic tweets. The values gradually decreased towards the weekend after reaching their peak in the middle of the week. Emoji could be a complementary behaviour for the expression of negative emotions that users employ more to compensate for the reduced use of text. That is, users still express their negative emotions in the middle of the week, but they do it with emojis rather than words.

Interestingly, similarities in pattern shape could be found in some cases in the shape of circadian and circaseptan patterns: in the textual analysis, the expression of negative emotions follows a U-shaped pattern in both datasets and using all three instruments for both circadian and circaseptan patterns.

Circadian patterns in positive emotions featured similarities in their shape between the LIWC and the VADER in both datasets. They decreased from midnight until the early morning which is when they increased until approximately 10.00 before then decreasing again until 14.00. From 14.00 onwards, the expression of positive emotions increased sharply until midnight, in line with previous studies using LIWC only [30]. Such daily rhythm can be explained by the organization of the working day: positive emotions might start decreasing when work stress begins to have an impact on our well-being and start increasing again when we are able to plan future scenarios and activities after the working hours.

Positive emotions followed similar circaseptan patterns in the LIWC, VADER, emoji—the Hu & Liu for Other topic tweets only, showing a gradual to sharp decrease from Sunday to Thursday and a gradual to sharp increase from Thursday to Saturday. They started decreasing on Sunday before the actual beginning of the working week. This occurred in both, Self-referencing and Other topic tweets. A comparable rhythm has been previously reported in Wang *et al*. [47]. However, in Wang *et al*., positive emotions began increasing on Tuesday, therefore, our analysis shows a 36–48 h delay in the commencement of the increasing trend. A similar pattern was observed in the expression of positive emotion in Other topic tweets in Hu & Liu. As the weekend approached, people expressed more positive emotions on Twitter. However, this pattern changed on Sunday, being in line with the progressive emotional discomfort experienced by people on Sunday afternoons known as the Sunday Blues [86].

The Hu & Liu-sentiment dimension and the VADER-compound dimension featured similar circadian patterns in both datasets. Both dimensions increased markedly from midnight to 8.00 and then decreased sharply from 8.00 until midnight which were opposite from the LIWC (Affect dimension) and the emoji sentiment dimension. The same was observed for circaseptan patterns where VADER-compound and Hu & Liu-sentiment slightly increased from Sunday to Wednesday, moderately decreased from Wednesday to Friday and then increased until Saturday.

Circadian patterns were similar in Self-referencing tweets and Other topic tweets for neutral emotions in VADER. Their expression increased sharply from midnight to 8.00 and markedly decreased from 8.00 to midnight. In emoji, the circadian pattern of neutral emotions featured slightly different shapes from the VADER. The same could be observed between datasets. While neutral emotions in Self-referencing tweets in emoji gradually increased from midnight to 15.00 and then decreased sharply, in Other topic tweets, they markedly increased from midnight to 10.00, then remained stable until 15.00 to sharply decrease from 15.00 to midnight. Circaseptan patterns were similar in Self-referencing and Other topic tweets in VADER. Neutral emotions increased from Sunday to Wednesday and then decreased until Saturday. By contrast, in emoji coding, neutral emotions slightly decreased from Sunday to Tuesday and then gradually increased until Saturday.

### 4.2. Similarities and differences in pattern amplitude

The positive emotion dimensions of all instruments featured circadian pattern amplitude differences between Self-referencing tweets and Other topic tweets. In all cases, the amplitude was larger in Other topic tweets. Similarly, circaseptan pattern amplitudes in positive emotions were larger in Other topic tweets than Self-referencing tweets, except in the case of emoji coding. The amplitude of circadian patterns in the negative emotions and composite dimensions was roughly similar between datasets for all instruments except emoji coding (larger amplitude in Other topic tweets). Larger amplitudes could be observed in circaseptan patterns in negative emotions in Self-referencing tweets for all instruments but the LIWC. The amplitude of circaseptan patterns in the composite dimensions was larger in Other topic tweets for LIWC-affect and VADER-compound, but larger in Self-referencing tweets for the Hu & Liu-sentiment dimensions and the emoji sentiment dimension. The neutral emotional dimension did not differ in circadian and circaseptan pattern amplitude for the VADER, but a larger (smaller) amplitude was observed for emoji coding in Other topic tweets for circadian (circaseptan) patterns. Finally, based on visual inspection, circadian patterns in the positive dimension of all tools are larger in Other topic tweets, but a larger *F-*value—suggesting a larger effect—was found in Self-referencing tweets for the VADER in the positive dimension (all other *F*-values for the positive dimensions in circadian patterns were larger in Self-referencing tweets corresponding to the visual analysis). The same thing can be said for circaseptan patterns in the positive dimension again.

### 4.3. Overall findings

The most important differences with regard to each comparison relate to: (i) differences in the amplitude of most circadian and circaseptan patterns in the comparison between Self-referencing tweets and Other topic tweets and some differences in pattern shape (e.g. Hu & Liu-positive, circaseptan pattern) and differences in the magnitude of *F*-values for each compared dimension but one-emoji-positive circadian, (ii) marked differences in the positive dimensions for the Hu & Liu in comparison with the other textual analysis tools, and (iii) the circaseptan patterns in the negative dimension in the analysis of emojis (lowest values during the weekend—in both datasets), which is opposite to those observed in all textual analysis tools (highest values during the weekend) and thereby displaying the complementarity of emotional expression in emojis and text.

The different tools correlated strongly with one another in their respective dimensions (positive and negative notably) but still featured sometimes different patterns. This raises the question of the dependence of findings of past research upon the tool of predilection (LIWC). Further studies should systematically investigate whether such differences are observed, and if so whether they actually mean that the different tools measure different aspect of the mentioned constructs, despite the high observed correlations between different measures of supposedly same constructs. Further, the reported study is also the first to statistically examine linear and nonlinear circadian and circaseptan patterns of PA and NA using mixed-model regression thereby partitioning out participant variance and allowing for more robust results.

### 4.4. Relevance and implications of findings

Understanding temporal patterns of associations with positive and negative emotions in tweets can contribute to the design of socio-technical systems aimed at supporting the emotional well-being of individuals through social media platforms in temporally relevant fashion. Such interventions could also foster the network of individuals who feel isolated. Indeed, positive mood is often associated with better health and higher subjective well-being [87,88]. People who reported positive mood in self-reports may also have stronger social networks [89]. In the workplace, positive mood did predict positive emotional contagion between workers and clients [87]. Individuals with negative mood are more likely to report being less physically active [90], poor health [91], presented delays in wound healings [92], worsening prolonged or chronic illnesses [93] and to experience less success in the adherence of treatment for illnesses [94]. Delayed PA phases have also been consistently associated with symptoms of depression [95]. Further, emotions can propagate from individual to individual, in face-to-face interactions as well as in the social media [38]. Interventions designed to support positive mood in the social media at the relevant time of the day, or days of the week—i.e. when positive (negative) expressed emotions are lower (higher)—could help alleviate negative consequences of low mood.

The differences in amplitude between Self-referencing tweets and Other topic tweets support our methodological decision and open up a new avenue for investigation more focused on the careful determination of what lexical, syntactic and iconic features of tweets may provide better answers to our research questions. This important aspect has been largely ignored in previous studies using comparable methodologies. Large datasets collected from Twitter are essential for tracking changes in the emotional states of a population over time. However, future studies should also pay closer attention to the qualities of such Twitter messages. That is, the emphasis should not only be on the collection of billions of tweets but also, and more importantly, on collecting tweets that are relevant for our research questions. Linguistic contexts affect word meanings; therefore, it is crucial to develop search criteria and automatic coding tools that could capture relevant features of Twitter messages beyond the mere lexical level (i.e. emotion words).

### 4.5. Limitations

Our search strategy (*I am* tweets) was aimed at increasing the likelihood that the content of the tweets related to the individual. However, this might have indirectly resulted in an oversampling of tweets produced by depressed individuals—who use first-person personal pronouns to a greater extent than non-depressed individuals [96]. Although our collection procedure did not allow us to ensure that people worked during traditional office hours (9.00–17.00), the period of data collection (the month of September) minimized the risk of holidays for the majority of participants. Moreover, our study was not designed to distinguish between evening-type and morning-type individuals with more typical work schedules. Further studies using content-coded tweets should investigate whether different types of individuals show different emotional trajectories throughout the day.

## 5. Conclusion

Our study is the first to focus on several relevant but overlooked comparisons in temporal patterns of emotional expression in tweets. First, we compared such patterns in Self-referencing tweets and Other topic tweets. Second, we compared results using different textual analysis instruments and third, we compared textual analyses with the analysis of emojis featured in tweets. Our results have shown similarities and differences in circadian and circaseptan patterns in all comparisons. This warrants further investigation.

We have discussed our findings from the perspective of the literature on emotions and well-being. Further research should also focus on how to use knowledge stemming from the study of temporal patterns in emotion to support individuals, notably through the social media, at the difficult moments of the day and the week which feature lower mood, and the conditions (e.g. users consent among others) under which such support is ethically acceptable.

## Data accessibility

The coded data are made available at https://osf.io/4c7kd/. Complying with the developer licence granted by Twitter, the tweets identifiers for this study are made available as well but not the textual content of the tweets. The tweets can be downloaded directly from Twitter using these identifiers.
