# Supplementary material for: Twitter, time and emotions
Source: R Soc Open Sci. 2021 May 26;8(5):201900. doi: 10.1098/rsos.201900 (PMC8150047; doi:10.1098/rsos.201900)
Supplement: Supplementary file 1 [file rsos201900_review_history.pdf]

THE ROYAL SOCIETY
PUBLISHING

ROYAL SOCIETY
OPEN SCIENCE

Twitter, time and emotions

Eric Mayor and Lucas M. Bietti

Article citation details
R. Soc. open sci. **8**: 201900.
http://dx.doi.org/10.1098/rsos.201900

Review timeline

Original submission:	6 October 2019
1st revised submission:	17 March 2020
2nd revised submission:	23 October 2020
3rd revised submission:	14 April 2021
Final acceptance:	6 May 2021

Note: Reports are unedited and appear as submitted by the referee. The review history appears in chronological order.

Review History

RSOS-191735.R0 (Original submission)

Review form: Reviewer 1

Is the manuscript scientifically sound in its present form?
Yes

Are the interpretations and conclusions justified by the results?
No

Is the language acceptable?
No

Do you have any ethical concerns with this paper?
No

Have you any concerns about statistical analyses in this paper?
No

Recommendation?
Major revision is needed (please make suggestions in comments)

Reports © 2021 The Reviewers; Decision Letters © 2021 The Reviewers and Editors; Responses © 2021 The Reviewers, Editors and Authors. Published by the Royal Society under the terms of the Creative Commons Attribution License http://creativecommons.org/licenses/by/4.0/, which permits unrestricted use, provided the original author and source are credited

Comments to the Author(s)
The article repeats previously conducted and very well known studies, about circadian and circaseptan variation of LIWC indicators. Individual variation for LIWC indicators was reported and shown in reference [13], contrary to what the authors suggest, as well as in a companion paper Dzogang F, Lightman S, Cristianini N. Circadian mood variations in Twitter content. Brain Neurosci Adv. 2017; 1(1). (Original DOI: 10.1177/2398212817744501)

The authors should emphasize that their contribution here is in repeating a known study on a different population, while still finding compatible results, which is important of course. Study [13] and the one listed above analysed each LIWC indicator on tweets collected every 15 mins in the UK for years. This study analyses the same LIWC indicators on tweets collected every 30 minutes in the US for a month. It is good that the overall behaviour is found again, despite the big difference in sampling - which could well have been a complete game changer - that only self-referential tweets are included in this study. Very interesting indeed, no need at all to downplay the work of those who conducted the first studies.

It is however not clear at all the meaning of studying how the correlation between signals changes with time - this is not explained well neither in the method, nor in its implication.

Review form: Reviewer 2

Is the manuscript scientifically sound in its present form?
Yes

Are the interpretations and conclusions justified by the results?
Yes

Is the language acceptable?
Yes

Do you have any ethical concerns with this paper?
Yes

Have you any concerns about statistical analyses in this paper?
No

Recommendation?
Major revision is needed (please make suggestions in comments)

Comments to the Author(s)
This paper confirms findings in 2 papers by the Cristianini group (and they do not cite the first one in Brain Neurosci Adv. 2017 Jan 1). The value of the current paper is that as before using using self-referring tweets (in a different country-the US as opposed to the UK) they still have the same findings. It is important to recognise however that they only collect for 30 days , rather than the 4 years in Cristianini's work.

I also worry about their claim that their contribution was to look at ALL indicators for the first time. They do not seem to have noticed this published figure!
https://doi.org/10.1371/journal.pone.0197002.g002

Review form: Reviewer 3

Is the manuscript scientifically sound in its present form?
Yes

Are the interpretations and conclusions justified by the results?
No

Is the language acceptable?
Yes

Do you have any ethical concerns with this paper?
No

Have you any concerns about statistical analyses in this paper?
Yes

Recommendation?
Reject

Comments to the Author(s)
The authors perform an analysis of daily and weekly variations of affective indicators related to a set of 64 LIWC factors over a set of tweets that contain personal, self-referential statements ("I am..."). Their findings indicate a wide variety of LIWC factors in the content of the set of tweets showing particular patterns of variation across a daily or weekly cycle.

In this work the authors restrict their analysis to tweets that are explicitly self-referential (they contain a variation of the statement "I am ...") and examine the relations between daily and weekly fluctuations in positive and negative emotions relative to 64 coding categories included in the LIWC tool.

The results show a plethora of statistically significant variations across those factors. The conclusions drawn from this study are not clear other than that "there exist variations" in LIWC categories associated with changes in positive and negative emotions.

I can not recommend this manuscript for publication in its present state for the following reasons:

1) The analysis is competently executed but I see major issues with the scientific rationale for this study. In lines 35 to 54 the authors explain that previous studies suffer from "important" limitations, namely that "The patterns of variations of each LIWC category were not examined (except for positive and negative emotions that were also investigated independently from the factors); and ii) The temporal patterns of associations between emotional categories and other LIWC categories were not clear because they have only been explored relying on the aggregation of dimensions [6, 13].".

Why is this considered an important limitation? In other words, other than that it hasn't been done, why should it be done?

In lines 50-54, the authors state they examined: "i) the existence of variation in expressed emotions in self-referring tweets through time, which constitutes a replication of various studies with the innovation of focusing on self-referring tweets". This is indeed a replication of many previous studies, but is the limitation to self-referential tweets a scientifically necessary innovation that warrants publication of this manuscript?

Furthermore, line 53: "ii) The existence of variation in other relevant LWIC categories... and iii) "the association of day... and week polynomials with the correlations between positive and negative emotions with other relevant LIWC-coded categories", is scientifically relevant only if one assumes there is a unique scientific knowledge gap with respect to specifically these LIWC categories. Why should the categories employed by a given text analysis tool serve as a scientific justification for a given analysis?

The following list of hypotheses seems to be construed to match what the authors already did, namely run LIWC over a set of tweets, not a scientific rationale to resolve an important knowledge gap in this field. The focus on "vary as a function of polynomials" also strikes me as odd. Why specifically a polynomial? I am not aware of an urgent or important knowledge gap in the literature specifically with respect to whether "proportion of content related to positive emotions <a LIWC category?>" does or does not "varies as a function of a polynomial of hour and day of the week."

2) the authors do carefully compensate their statistical significance criterion for the exceptionally high number of repeated tests: "a maximum N = 160 counties * 4 weeks * 7 days * 24 hours = 107,520 correlations for each considered variable (4.193 millions in total)", and "we performed a total of 390 tests: 3915 categories * (5 polynomials for day + 5 polynomials for hour)" but this leads to a situation where one has to worry about the "false discovery rate" where a few factors will inevitably return a statistically significant result. This is obvious from the discussions of results in sections 4.1, 4.2, 4.3, 4.4. which are essentially long lists of all LIWC categories and indicators that registered a statistically significant correlations across 12 patterns and 64 LIWC categories. Again, one has to wonder about the scientific relevance of these specific LIWC categories and what conclusions can be drawn from the fact that that the authors found polynomials that describes the correlation between daily and weekly emotional variations with those categories, etc.

Decision letter (RSOS-191735.R0)

21-Feb-2020

Dear Dr Bietti,

The editors assigned to your paper ("Twitter, Time and Emotions") have now received comments from reviewers. We would like you to revise your paper in accordance with the referee and Associate Editor suggestions which can be found below (not including confidential reports to the Editor). Please note this decision does not guarantee eventual acceptance.

Please submit a copy of your revised paper before 15-Mar-2020. Please note that the revision deadline will expire at 00.00am on this date. If we do not hear from you within this time then it will be assumed that the paper has been withdrawn. In exceptional circumstances, extensions may be possible if agreed with the Editorial Office in advance. We do not allow multiple rounds of revision so we urge you to make every effort to fully address all of the comments at this stage. If deemed necessary by the Editors, your manuscript will be sent back to one or more of the original reviewers for assessment. If the original reviewers are not available, we may invite new reviewers.

To revise your manuscript, log into http://mc.manuscriptcentral.com/rsos and enter your Author Centre, where you will find your manuscript title listed under "Manuscripts with

Decisions." Under "Actions," click on "Create a Revision." Your manuscript number has been appended to denote a revision. Revise your manuscript and upload a new version through your Author Centre.

When submitting your revised manuscript, you must respond to the comments made by the referees and upload a file "Response to Referees" in "Section 6 - File Upload". Please use this to document how you have responded to the comments, and the adjustments you have made. In order to expedite the processing of the revised manuscript, please be as specific as possible in your response.

In addition to addressing all of the reviewers' and editor's comments please also ensure that your revised manuscript contains the following sections as appropriate before the reference list:

• Ethics statement (if applicable)
If your study uses humans or animals please include details of the ethical approval received, including the name of the committee that granted approval. For human studies please also detail whether informed consent was obtained. For field studies on animals please include details of all permissions, licences and/or approvals granted to carry out the fieldwork.

• Data accessibility
It is a condition of publication that all supporting data are made available either as supplementary information or preferably in a suitable permanent repository. The data accessibility section should state where the article's supporting data can be accessed. This section should also include details, where possible of where to access other relevant research materials such as statistical tools, protocols, software etc can be accessed. If the data have been deposited in an external repository this section should list the database, accession number and link to the DOI for all data from the article that have been made publicly available. Data sets that have been deposited in an external repository and have a DOI should also be appropriately cited in the manuscript and included in the reference list.

If you wish to submit your supporting data or code to Dryad (http://datadryad.org/), or modify your current submission to dryad, please use the following link:
http://datadryad.org/submit?journalID=RSOS&manu=RSOS-191735

• Competing interests
Please declare any financial or non-financial competing interests, or state that you have no competing interests.

• Authors' contributions
All submissions, other than those with a single author, must include an Authors' Contributions section which individually lists the specific contribution of each author. The list of Authors should meet all of the following criteria; 1) substantial contributions to conception and design, or acquisition of data, or analysis and interpretation of data; 2) drafting the article or revising it critically for important intellectual content; and 3) final approval of the version to be published.

All contributors who do not meet all of these criteria should be included in the acknowledgements.

We suggest the following format:
AB carried out the molecular lab work, participated in data analysis, carried out sequence alignments, participated in the design of the study and drafted the manuscript; CD carried out the statistical analyses; EF collected field data; GH conceived of the study, designed the study,

coordinated the study and helped draft the manuscript. All authors gave final approval for publication.

• Acknowledgements
Please acknowledge anyone who contributed to the study but did not meet the authorship criteria.

• Funding statement
Please list the source of funding for each author.

Once again, thank you for submitting your manuscript to Royal Society Open Science and I look forward to receiving your revision. If you have any questions at all, please do not hesitate to get in touch.

Kind regards,
Andrew Dunn
Royal Society Open Science Editorial Office
Royal Society Open Science
openscience@royalsociety.org

on behalf of Dr Christina Demski (Associate Editor) and Essi Viding (Subject Editor)
openscience@royalsociety.org

Associate Editor's comments (Dr Christina Demski):
Associate Editor: 1
Comments to the Author:
Two of the reviewers have evaluated the paper as scientifically sound and of value, although both raise concerns with regards to the overall framing of this manuscript and how it relates to previous literature. Please address comments from all three reviewers if you choose to make revisions to the manuscript.

Comments to Author:

Reviewers' Comments to Author:
Reviewer: 1

Comments to the Author(s)
The article repeats previously conducted and very well known studies, about circadian and circaseptan variation of LIWC indicators. Individual variation for LIWC indicators was reported and shown in reference [13], contrary to what the authors suggest, as well as in a companion paper Dzogang F, Lightman S, Cristianini N. Circadian mood variations in Twitter content. Brain Neurosci Adv. 2017; 1(1). (Original DOI: 10.1177/2398212817744501)

The authors should emphasize that their contribution here is in repeating a known study on a different population, while still finding compatible results, which is important of course. Study [13] and the one listed above analysed each LIWC indicator on tweets collected every 15 mins in the UK for years. This study analyses the same LIWC indicators on tweets collected every 30 minutes in the US for a month. It is good that the overall behaviour is found again, despite the big difference in sampling - which could well have been a complete game changer - that only self-referential tweets are included in this study. Very interesting indeed, no need at all to downplay the work of those who conducted the first studies.

It is however not clear at all the meaning of studying how the correlation between signals changes with time - this is not explained well neither in the method, nor in its implication.

Reviewer: 2

Comments to the Author(s)
This paper confirms findings in 2 papers by the Cristianini group (and they do not cite the first one in Brain Neurosci Adv. 2017 Jan 1). The value of the current paper is that as before using using self-referring tweets (in a different country-the US as opposed to the UK) they still have the same findings. It is important to recognise however that they only collect for 30 days , rather than the 4 years in Cristianini's work.

I also worry about their claim that their contribution was to look at ALL indicators for the first time. They do not seem to have noticed this published figure!
https://doi.org/10.1371/journal.pone.0197002.g002

Reviewer: 3

Comments to the Author(s)
The authors perform an analysis of daily and weekly variations of affective indicators related to a set of 64 LIWC factors over a set of tweets that contain personal, self-referential statements ("I am..."). Their findings indicate a wide variety of LIWC factors in the content of the set of tweets showing particular patterns of variation across a daily or weekly cycle.

In this work the authors restrict their analysis to tweets that are explicitly self-referential (they contain a variation of the statement "I am ...") and examine the relations between daily and weekly fluctuations in positive and negative emotions relative to 64 coding categories included in the LIWC tool.

The results show a plethora of statistically significant variations across those factors. The conclusions drawn from this study are not clear other than that "there exist variations" in LIWC categories associated with changes in positive and negative emotions.

I can not recommend this manuscript for publication in its present state for the following reasons:

1) The analysis is competently executed but I see major issues with the scientific rationale for this study. In lines 35 to 54 the authors explain that previous studies suffer from "important" limitations, namely that "The patterns of variations of each LIWC category were not examined (except for positive and negative emotions that were also investigated independently from the factors); and ii) The temporal patterns of associations between emotional categories and other LIWC categories were not clear because they have only been explored relying on the aggregation of dimensions [6, 13].".

Why is this considered an important limitation? In other words, other than that it hasn't been done, why should it be done?

In lines 50-54, the authors state they examined: "i) the existence of variation in expressed emotions in self-referring tweets through time, which constitutes a replication of various studies with the innovation of focusing on self-referring tweets". This is indeed a replication of many previous studies, but is the limitation to self-referential tweets a scientifically necessary innovation that warrants publication of this manuscript?

Furthermore, line 53: "ii) The existence of variation in other relevant LWIC categories... and iii) "the association of day... and week polynomials with the correlations between positive and negative emotions with other relevant LIWC-coded categories", is scientifically relevant only if one assumes there is a unique scientific knowledge gap with respect to specifically these LIWC categories. Why should the categories employed by a given text analysis tool serve as a scientific justification for a given analysis?

The following list of hypotheses seems to be construed to match what the authors already did, namely run LIWC over a set of tweets, not a scientific rationale to resolve an important knowledge gap in this field. The focus on "vary as a function of polynomials" also strikes me as odd. Why specifically a polynomial? I am not aware of an urgent or important knowledge gap in the literature specifically with respect to whether "proportion of content related to positive emotions <a LIWC category?>" does or does not "varies as a function of a polynomial of hour and day of the week."

2) the authors do carefully compensate their statistical significance criterion for the exceptionally high number of repeated tests: "a maximum N = 160 counties * 4 weeks * 7 days * 24 hours = 107,520 correlations for each considered variable (4.193 millions in total)", and "we performed a total of 390 tests: 3915 categories * (5 polynomials for day + 5 polynomials for hour)" but this leads to a situation where one has to worry about the "false discovery rate" where a few factors will inevitably return a statistically significant result. This is obvious from the discussions of results in sections 4.1, 4.2, 4.3, 4.4. which are essentially long lists of all LIWC categories and indicators that registered a statistically significant correlations across 12 patterns and 64 LIWC categories. Again, one has to wonder about the scientific relevance of these specific LIWC categories and what conclusions can be drawn from the fact that that the authors found polynomials that describes the correlation between daily and weekly emotional variations with those categories, etc.

Author's Response to Decision Letter for (RSOS-191735.R0)

See Appendix A.

RSOS-191735.R1 (Revision)

Review form: Reviewer 1

Is the manuscript scientifically sound in its present form?
No

Are the interpretations and conclusions justified by the results?
No

Is the language acceptable?
No

Do you have any ethical concerns with this paper?
No

Have you any concerns about statistical analyses in this paper?
Yes

Recommendation?
Reject

Comments to the Author(s)
Dear Authors,
your chosen topic is of real interest, but this article does not have any new message for the reader. Besides replicating known studies in slightly different settings, there is no evidence that those settings are any better, there is no discussion of lesson learnt from the differences, and the two statistical tests are not justified, badly explained, and lead to no insight. My advice is: start from what you want to find out, then work your way backwards to the data and analysis. I really wanted to learn something new and clear from those experiments, but I could not.

Review form: Reviewer 2

Is the manuscript scientifically sound in its present form?
Yes

Are the interpretations and conclusions justified by the results?
Yes

Is the language acceptable?
Yes

Do you have any ethical concerns with this paper?
No

Have you any concerns about statistical analyses in this paper?
No

Recommendation?
Accept as is

Comments to the Author(s)
My issues have been addressed by the authors

Decision letter (RSOS-191735.R1)

We hope you are keeping well at this difficult and unusual time. We continue to value your support of the journal in these challenging circumstances. If Royal Society Open Science can assist you at all, please don't hesitate to let us know at the email address below.

Dear Dr Bietti:

Manuscript ID RSOS-191735.R1 entitled "Twitter, Time and Emotions" which you submitted to Royal Society Open Science, has been reviewed. The comments from reviewer(s) are included at the bottom of this letter.

In view of the criticisms of the reviewer(s), I must decline the manuscript for publication in Royal Society Open Science at this time. However, a new manuscript may be submitted which takes into consideration these comments.

Please note that resubmitting your manuscript does not guarantee eventual acceptance, and that your resubmission will be subject to re-review by the reviewer(s) before a decision is rendered.

You will be unable to make your revisions on the originally submitted version of your manuscript. Instead, revise your manuscript using a word processing program and save it on your computer.

Once you have revised your manuscript, go to https://mc.manuscriptcentral.com/rsos and login to your Author Center. Click on "Manuscripts with Decisions," and then click on "Create a Resubmission" located next to the manuscript number. Then, follow the steps for resubmitting your manuscript.

You may also click the below link to start the resubmission process (or continue the process if you have already started your resubmission) for your manuscript. If you use the below link you will not be required to login to ScholarOne Manuscripts.

*** PLEASE NOTE: This is a two-step process. After clicking on the link, you will be directed to a webpage to confirm. ***

https://mc.manuscriptcentral.com/rsos?URL_MASK=247549333a3f4d1bb9a4c61113fd03a5

Because we are trying to facilitate timely publication of manuscripts submitted to Royal Society Open Science, your resubmitted manuscript should be submitted by 29-Oct-2020. If you are unable to submit by this date please contact the Editorial Office for options.

I look forward to a resubmission.

Kind regards,
Anita Kristiansen
Editorial Coordinator

Royal Society Open Science
openscience@royalsociety.org

on behalf of Dr Christina Demski (Associate Editor)
openscience@royalsociety.org

Associate Editor Comments to Author (Dr Christina Demski):

While it is evident that some of the reviewer concerns have now been addressed, unfortunately one reviewer is not satisfied with the scientific rationale of the paper. This is a concern that has now been raised by two reviewers and means we are not able to accept the manuscript in the present form. However, if you would like to resubmit at a later stage, this option is open to you.

Reviewer comments to Author:
Reviewer: 2
Comments to the Author(s)
My issues have been addressed by the authors

Reviewer: 1
Comments to the Author(s)
Dear Authors,
your chosen topic is of real interest, but this article does not have any new message for the reader. Besides replicating known studies in slightly different settings, there is no evidence that those settings are any better, there is no discussion of lesson learnt from the differences, and the two statistical tests are not justified, badly explained, and lead to no insight. My advice is: start from what you want to find out, then work your way backwards to the data and analysis. I really wanted to learn something new and clear from those experiments, but I could not.

Author's Response to Decision Letter for (RSOS-191735.R1)

See Appendix B.

Decision letter (RSOS-201900.R0)

We hope you are keeping well at this difficult and unusual time. We continue to value your support of the journal in these challenging circumstances. If Royal Society Open Science can assist you at all, please don't hesitate to let us know at the email address below.

Dear Dr Bietti

On behalf of the Editors, we are pleased to inform you that your Manuscript RSOS-201900 "Twitter, Time and Emotions" has been accepted for publication in Royal Society Open Science subject to minor revision in accordance with the referees' reports. Please find the referees' comments along with any feedback from the Editors below my signature.

We invite you to respond to the comments and revise your manuscript. Below the referees' and Editors' comments (where applicable) we provide additional requirements. Final acceptance of your manuscript is dependent on these requirements being met. We provide guidance below to help you prepare your revision.

Please submit your revised manuscript and required files (see below) no later than 7 days from today's (ie 26-Mar-2021) date. Note: the ScholarOne system will 'lock' if submission of the revision is attempted 7 or more days after the deadline. If you do not think you will be able to meet this deadline please contact the editorial office immediately.

Please note article processing charges apply to papers accepted for publication in Royal Society Open Science (https://royalsocietypublishing.org/rsos/charges). Charges will also apply to papers transferred to the journal from other Royal Society Publishing journals, as well as papers submitted as part of our collaboration with the Royal Society of Chemistry

(https://royalsocietypublishing.org/rsos/chemistry). Fee waivers are available but must be requested when you submit your revision (https://royalsocietypublishing.org/rsos/waivers).

Thank you for submitting your manuscript to Royal Society Open Science and we look forward to receiving your revision. If you have any questions at all, please do not hesitate to get in touch.

Best regards,
Lianne Parkhouse
Editorial Coordinator
Royal Society Open Science
openscience@royalsociety.org

on behalf of Dr Gina Grimshaw (Associate Editor) and Essi Viding (Subject Editor)
openscience@royalsociety.org

Associate Editor Comments to Author (Dr Gina Grimshaw):

I was not the original AE on this manuscript. However, I have read the manuscript carefully along with previous decisions and author responses. I appreciate you have made substantial revisions to the manuscript, and believe that you have now provided good scientific rationale for your primary analyses (self-relevant vs. other tweets; text vs. emojis; and comparisons across coding systems). I recommend acceptance of the manuscript pending some relatively minor amendments.

1. LIWC and VADER are acronyms for well-known coding systems. However, for non-expert readers it would be useful to provide the full names of these systems on first mention. Moreover, given that one goal of the manuscript is to compare systems, it would be useful to highlight how the systems differ conceptually. This could be done briefly when the measures are described in the Method, and referred to in the Discussion.

2. I agree that the mixed-model regression are the appropriate way to analyse the data, but one problem with the method is that it can be over-powered (particularly when used with such large samples), with the result that everything is significant. I think using the F values for comparison of conditions is a reasonable way to compare effects, but what of the different polynomials (linear to quintic) which are also all signficant? Table 2 provides only the F value for the complete model but not the trends, and so it is not possible to know if the trends differ in their relative importance to the overall pattern. Are the B values (provided in text) interpretable? Moreover, you then provide qualitative descriptions of the patterns (e.g., the proportion of PA might peak at midnight, decline until 8 am, and then slowly rise again...). If the descriptions are so qualitative, what do the individual polynomial trends contribute? Readers could use some guidance here on how to interpret the polynomial effects.

3. The files on OSF provide the unique identifiers, location, time, and date of each tweet. However, for the purposes of reproducing your findings, it would be useful to provide the values of each dimension according to each coding system, and the code for your statistical analyses of that data. I don't see that these data should violate any of twitter's licensing requirements.

===PREPARING YOUR MANUSCRIPT===

Your revised paper should include the changes requested by the referees and Editors of your manuscript. You should provide two versions of this manuscript and both versions must be provided in an editable format:

one version identifying all the changes that have been made (for instance, in coloured highlight, in bold text, or tracked changes);
a 'clean' version of the new manuscript that incorporates the changes made, but does not highlight them. This version will be used for typesetting.
Please ensure that any equations included in the paper are editable text and not embedded images.

Please ensure that you include an acknowledgements' section before your reference list/bibliography. This should acknowledge anyone who assisted with your work, but does not qualify as an author per the guidelines at https://royalsociety.org/journals/ethics-policies/openness/.

While not essential, it will speed up the preparation of your manuscript proof if you format your references/bibliography in Vancouver style (please see https://royalsociety.org/journals/authors/author-guidelines/#formatting). You should include DOIs for as many of the references as possible.

If you have been asked to revise the written English in your submission as a condition of publication, you must do so, and you are expected to provide evidence that you have received language editing support. The journal would prefer that you use a professional language editing service and provide a certificate of editing, but a signed letter from a colleague who is a native speaker of English is acceptable. Note the journal has arranged a number of discounts for authors using professional language editing services (https://royalsociety.org/journals/authors/benefits/language-editing/).

===PREPARING YOUR REVISION IN SCHOLARONE===

To revise your manuscript, log into https://mc.manuscriptcentral.com/rsos and enter your Author Centre - this may be accessed by clicking on "Author" in the dark toolbar at the top of the page (just below the journal name). You will find your manuscript listed under "Manuscripts with Decisions". Under "Actions", click on "Create a Revision".

Attach your point-by-point response to referees and Editors at Step 1 'View and respond to decision letter'. This document should be uploaded in an editable file type (.doc or .docx are preferred). This is essential.

Please ensure that you include a summary of your paper at Step 2 'Type, Title, & Abstract'. This should be no more than 100 words to explain to a non-scientific audience the key findings of your research. This will be included in a weekly highlights email circulated by the Royal Society press office to national UK, international, and scientific news outlets to promote your work.

At Step 3 'File upload' you should include the following files:
-- Your revised manuscript in editable file format (.doc, .docx, or .tex preferred). You should upload two versions:
1) One version identifying all the changes that have been made (for instance, in coloured highlight, in bold text, or tracked changes);
2) A 'clean' version of the new manuscript that incorporates the changes made, but does not highlight them.
-- An individual file of each figure (EPS or print-quality PDF preferred [either format should be produced directly from original creation package], or original software format).
-- An editable file of each table (.doc, .docx, .xls, .xlsx, or .csv).
-- An editable file of all figure and table captions.
Note: you may upload the figure, table, and caption files in a single Zip folder.

-- Any electronic supplementary material (ESM).
-- If you are requesting a discretionary waiver for the article processing charge, the waiver form must be included at this step.
-- If you are providing image files for potential cover images, please upload these at this step, and inform the editorial office you have done so. You must hold the copyright to any image provided.
-- A copy of your point-by-point response to referees and Editors. This will expedite the preparation of your proof.

At Step 6 'Details & comments', you should review and respond to the queries on the electronic submission form. In particular, we would ask that you do the following:
-- Ensure that your data access statement meets the requirements at https://royalsociety.org/journals/authors/author-guidelines/#data. You should ensure that you cite the dataset in your reference list. If you have deposited data etc in the Dryad repository, please only include the 'For publication' link at this stage. You should remove the 'For review' link.
-- If you are requesting an article processing charge waiver, you must select the relevant waiver option (if requesting a discretionary waiver, the form should have been uploaded at Step 3 'File upload' above).
-- If you have uploaded ESM files, please ensure you follow the guidance at https://royalsociety.org/journals/authors/author-guidelines/#supplementary-material to include a suitable title and informative caption. An example of appropriate titling and captioning may be found at https://figshare.com/articles/Table_S2_from_Is_there_a_trade-off_between_peak_performance_and_performance_breadth_across_temperatures_for_aerobic_scope_in_teleost_fishes_/3843624.

At Step 7 'Review & submit', you must view the PDF proof of the manuscript before you will be able to submit the revision. Note: if any parts of the electronic submission form have not been completed, these will be noted by red message boxes.

Author's Response to Decision Letter for (RSOS-201900.R0)

See Appendix C.

Decision letter (RSOS-201900.R1)

We hope you are keeping well at this difficult and unusual time. We continue to value your support of the journal in these challenging circumstances. If Royal Society Open Science can assist you at all, please don't hesitate to let us know at the email address below.

Dear Dr Bietti,

It is a pleasure to accept your manuscript entitled "Twitter, Time and Emotions" in its current form for publication in Royal Society Open Science. The comments of the reviewer(s) who reviewed your manuscript are included at the foot of this letter.

Please ensure that you send to the editorial office an editable version of your accepted manuscript, and individual files for each figure and table included in your manuscript. You can send these in a zip folder if more convenient. Failure to provide these files may delay the

processing of your proof. You may disregard this request if you have already provided these files to the editorial office.

You can expect to receive a proof of your article in the near future. Please contact the editorial office (openscience@royalsociety.org) and the production office (openscience_proofs@royalsociety.org) to let us know if you are likely to be away from e-mail contact -- if you are going to be away, please nominate a co-author (if available) to manage the proofing process, and ensure they are copied into your email to the journal.

Due to rapid publication and an extremely tight schedule, if comments are not received, your paper may experience a delay in publication.

Please see the Royal Society Publishing guidance on how you may share your accepted author manuscript at https://royalsociety.org/journals/ethics-policies/media-embargo/. After publication, some additional ways to effectively promote your article can also be found here https://royalsociety.org/blog/2020/07/promoting-your-latest-paper-and-tracking-your-results/.

Thank you for your fine contribution. On behalf of the Editors of Royal Society Open Science, we look forward to your continued contributions to the Journal.

Kind regards,
Anita Kristiansen
Editorial Coordinator

Royal Society Open Science
openscience@royalsociety.org

on behalf of Dr Gina Grimshaw (Associate Editor) and Essi Viding (Subject Editor)
openscience@royalsociety.org

Associate Editor Comments to Author (Dr Gina Grimshaw):
Comments to the Author:
Thank you for submitting these revisions to the manuscript. I believe that all concerns have now been addressed.

Follow Royal Society Publishing on Twitter: @RSocPublishing
Follow Royal Society Publishing on Facebook:
https://www.facebook.com/RoyalSocietyPublishing.FanPage/
Read Royal Society Publishing's blog:
https://royalsociety.org/blog/blogsearchpage/?category=Publishing

Appendix A

Dear Editor,

We thank you for your recent decision on our manuscript titled “Twitter, Time and Emotions” (ID RSOS-191735). We also thank the reviewers for their precious comments and suggestions, which have improved our contribution. Below we discuss these point by point, explaining how we implemented the suggested changes or clarified the relevant aspects in the manuscript.

We hope our manuscript is now ready to be accepted for publication and remain available to answer your potential questions.

Sincerely yours,
Eric Mayor and Lucas Bietti

Reviewer: 1

The article repeats previously conducted and very well-known studies, about circadian and circaseptan variation of LIWC indicators. Individual variation for LIWC indicators was reported and shown in reference [13], contrary to what the authors suggest, as well as in a companion paper Dzogang F, Lightman S, Cristianini N. Circadian mood variations in Twitter content. Brain Neurosci Adv. 2017; 1(1). (Original DOI: 10.1177/2398212817744501).

=> We have acknowledged that previous studies have found similar results (e.g., Dzongang et al., 2018 using diurnal variation indices) with different population and sample sizes. We have changed the text accordingly and have included the mentioned reference (Dzongang et al., 2017) in the discussion. We thank the reviewer for pointing this out.

The authors should emphasize that their contribution here is in repeating a known study on a different population, while still finding compatible results, which is important of course. Study [13] and the one listed above analysed each LIWC indicator on tweets collected every 15 mins in the UK for years. This study analyses the same LIWC indicators on tweets collected every 30 minutes in the US for a month. It is good that the overall behaviour is found again, despite the big difference in sampling - which could well have been a complete game changer - that only self-referential tweets are included in this study. Very interesting indeed, no need at all to downplay the work of those who conducted the first studies.

=> We have clarified that our study is in part a replication of previous studies. We now mention that our paper complements existing research (rather than downplaying their role). We acknowledge that the main novelty of the study relies on having analyzed self-referential tweets produced in the US (see pages 3 and 4). Focusing on self-referential tweets is important for the following two reasons: i) individuals recall better content related to themselves than content related to other people and ii) Self-reference is related to deeper processing, higher organization and elaboration of self-related material and its importance extends to self-appraisals. As the reviewer nicely points out, we think the successful replication (obtaining results similar as those of previous studies) is interesting per se. Yet, we believe our study offers other interesting contributions.

Reviewer: 2

Comments to the Author(s)
This paper confirms findings in 2 papers by the Cristianini group (and they do not cite the first one in Brain Neurosci Adv. 2017 Jan 1). The value of the current paper is that as before using self-referring tweets (in a different country-the US as opposed to the UK) they still have the same findings. It is important to recognise however that they only collect for 30 days, rather than the 4 years in Cristianini's work.

=> We have made clear that the main contribution of the paper is the focus on the self-referential tweets (see pages 2 and 3). We have also acknowledged that Dzongang et al (from Christiani's group) have obtained similar results with regards to diurnal variations of PA and NA using different data analyses. As Reviewer 2 mentioned, our findings also extend previous research as we have used a sample from a different population (US instead of UK). We now also describe the statistical advantage of the statistical approach we undertook in order to minimize county-level bias of statistical estimates. ("(…) our study is the first to statistically partial out variance lying at the level of the regions (counties) composing the territory in which the collected tweets originate, for the study of circadian (24-hour) and circaseptan (7-day) patterns of variation. This allows an improved estimation of model parameters and of statistical significance ").

We also worry about their claim that their contribution was to look at ALL indicators for the first time. They do not seem to have noticed this published figure!
https://doi.org/10.1371/journal.pone.0197002.g002

=> We now have explained that our study complements and extends the studies conducted notably by Christianini's group in the UK regarding H1 and H2. However, we believe we are the first to statistically examine linear and non-linear circadian and circaseptan patterns of PA and NA (Christianini's group provided heatmaps of circadian patterns and tested overall daily variation). We further note that: "we excluded categories from linguistic processes and spoken categories as they were considered less relevant because of the search query we employed: personal pronoun + present tense verb: I am and its variations. "

Reviewer: 3

Comments to the Author(s)

The authors perform an analysis of daily and weekly variations of affective indicators related to a set of 64 LIWC factors over a set of tweets that contain personal, self-referential statements ("I am..."). Their findings indicate a wide variety of LIWC factors in the content of the set of tweets showing particular patterns of variation across a daily or weekly cycle.

In this work the authors restrict their analysis to tweets that are explicitly self-referential (they contain a variation of the statement "I am ...") and examine the relations between daily and weekly fluctuations in positive and negative emotions relative to 64 coding categories included in the LIWC tool.

The results show a plethora of statistically significant variations across those factors. **The conclusions drawn from this study are not clear other than that "there exist variations" in LIWC categories associated with changes in positive and negative emotions.**

=> We made the contributions of our study clearer in the discussion: "Our study shows that 1) previous findings (e.g., [11]) relating to circadian (24-hour) and circaseptan (7-day) patterns in emotions also apply to self-referring tweets, 2) such patterns can be observed in other individual content categories, and in associations between content categories and expressed emotions; and 3) there exist more circadian and circaseptan trajectories than previously assumed in the literature (e.g. [13]), and different trajectories exist in associations of several individual LIWC categories with positive and negative affect as well." (also see following development). We mentioned in the discussion that these trajectories have been classified and described (results). We also specified that "(…) our study is the first to statistically partial out variance lying at the level of the regions (counties) composing the territory in which the collected tweets originate, for the study of circadian (24-hour) and circaseptan (7-day) patterns of variation. This allows an improved estimation of model parameters and of statistical significance."

I can not recommend this manuscript for publication in its present state for the following reasons:

1) The analysis is competently executed but I see **major issues** with the scientific rationale for this study. In lines 35 to 54 the authors explain that previous studies suffer from "important" limitations, namely that "The patterns of variations of each LIWC category were not examined (except for positive and negative emotions that were also investigated independently from the factors); and ii) The temporal patterns of associations between emotional categories and other LIWC categories were not clear because they have only been explored relying on the aggregation of dimensions [6, 13].".

Why is this considered an important limitation? In other words, other than that it hasn't been done, why should it be done?

=> We have adapted the relevant portion of our introduction (lines 35+ in the previous version of the manuscript – mentioned by R3) in line with the R3's comment above and other comments from R1 and R2:" [13] also computed, and provided heatmaps, of the diurnal variation in the LIWC categories. Complementing previous studies, we investigate i) The temporal patterns in LIWC categories and ii) The temporal patterns of associations between emotional categories and other LIWC categories individually [6, 13]. Both issues require investigation for different reasons. Regarding the first issue, it is certainly of interest to know whether the general findings of these authors hold in the specific case of each LIWC category. This is because the LIWC dictionary is not arbitrary but is based upon thorough psychological research aiming at identifying, testing and validating word categories assessing "basic emotional and cognitive dimensions often studied in social, health, and personality psychology" ([7], p. 7). It is important to understand how such dimensions vary through time individually rather than in an aggregated fashion because each category measures different and psychologically relevant meaning which is obfuscated by aggregation. The second issue relates to the understanding of patterns of associations of positive and negative emotions with the other constructs assessed in LIWC categories. Research considering emotions as a criterion variable is interested in their associations with different predictors (e.g., social support: Gallagher & Vella-Brodrick, 2008; physical activity: Pasco, Jacka, Williams, Brennan, Leslie & Berk, 2011). The notion that such effects can vary through time is extraneous to this field. Understanding temporal variation in emotions in relation to different psychological processes is interesting in its own right and more importantly because it puts into question such implicit assumption of temporal stability of such effects."

In lines 50-54, the authors state they examined: "i) the existence of variation in expressed emotions in self-referring tweets through time, which constitutes a replication of various studies with the innovation of focusing on self-referring tweets". This is indeed a replication of many previous studies, **but is the limitation to self-referential tweets a scientifically necessary innovation that warrants publication of this manuscript?**

=> As another reviewer wrote regarding the replication portion of this contribution (H1 and H2): should we, using only self-referential tweets in our analysis, have found patterns that differed from previous research (using undifferentiated tweets), this would have been a game changer. Indeed, most research on emotion using the LIWC implicitly considers the expression of emotion by individuals in their texts reflects their own emotions. Our results for H1 and H2 using self-referring tweets is convergent with past research on tweets in general, which seems to indicate that this is assumed correspondence is valid. Our study further tests H1 and H2 in a population different from other studies.

Furthermore, line 53: "ii) The existence of variation in other relevant LWIC categories... and iii) "the association of day... and week polynomials with the correlations between positive and negative emotions with other relevant LIWC-coded categories", **is scientifically relevant only if one assumes there is a unique scientific knowledge gap with respect to specifically these LIWC categories**.

Why should the categories employed by a given text analysis tool serve as a scientific justification for a given analysis?

=> Years of studies by Pennebaker and colleagues as well as others have shown the relevance of the dictionary categories of the LWIC to understand cognition and emotion as expressed in texts. As mentioned above "It is important to understand how such dimensions vary through time individually rather than in an aggregated fashion because each category measures different and psychologically relevant meaning which is obfuscated by aggregation." To our opinion, this justifies the use of the LIWC categories, because the LIWC is built to reflect psychologically relevant dimensions and the aim of this paper is to study their variations and associations. We further note that: "we excluded categories from linguistic processes and spoken categories as they were considered less relevant because of the search query we employed: personal pronoun + present tense verb: I am and its variations."

The following list of hypotheses seems to be construed to match what the authors already did, namely run LIWC over a set of tweets, not a scientific rationale to resolve an important knowledge gap in this field. The focus on "vary as a function of polynomials" also strikes me as odd. Why specifically a polynomial? I am **not aware of an urgent or important knowledge gap in the literature specifically with respect to whether "proportion of content related to positive emotions <a LIWC category?>" does or does not "varies as a function of a polynomial of hour and day of the week."**

=> We should not have allowed a confusion between our research questions and their conceptual operationalization. We apologize for this. We now clarify this in the manuscript by calling our hypotheses "operational hypotheses". The research questions we propose are quite similar to the rest of the literature for H1 and H2, which we believe they extend for H3 to H5. The question of whether the standard output of LIWC is a proportion of content or another measure is not crucial here as the reviewer indicates. Regarding the relevance of using different LIWC categories, we refer to our previous answer. By mentioning the relevance of using polynomials, the reviewer refers to our operationalization of the effect of Time. The use of polynomials is indeed an analytical choice. The use of several polynomials instead of e.g., one or two is relevant because examining only linear (polynomial of degree 1) or quadratic (polynomial of degree 2) functions would not account for the complexity that could be occur in the data. In other words, using simpler models (e.g., linear and quadratic effects only) would lead to unrealistically simple patterns of change. The approach we chose allows to model up to four turning points in the data and to reflect in a meaningful way the patterns in the variables under investigation, even though we acknowledge these could be more complex. Dzogang et al. (2018) have opted for another equally valid way (examination of cyclicity using Fast Fourier Transform) to model such complexity.

2) the authors do carefully compensate their statistical significance criterion for the exceptionally high number of repeated tests: "a maximum N = 160 counties * 4 weeks * 7 days * 24 hours = 107,520 correlations for each considered variable (4.193 millions in total)", and "we performed a total of 390 tests: 3915 categories * (5 polynomials for day + 5 polynomials for hour)" but this leads to a situation where one has to worry about the "false discovery rate" **where a few factors will inevitably return a statistically significant result.** This is obvious from the discussions of results in sections 4.1, 4.2, 4.3, 4.4. which are essentially long lists of all LIWC categories and indicators that registered a statistically significant correlations across 12 patterns and 64 LIWC categories. Again, one has to wonder about the **scientific relevance of these specific LIWC categories** and what conclusions can be drawn from the fact that that the authors found polynomials that describes the correlation between daily and weekly emotional variations with those categories, etc.

=> We are aware of the risk of obtaining more statistically significant results (Type I error) with increased number of tests. This is why we have used Bonferroni significance correction to adequately compensate the statistical significance threshold of each of the tests. Therefore the risk of Type I error in this study is the same or more conservative as in research using one or more statistical tests without compensating for multiple tests. The reviewer points out the relevance of the LIWC categories a third

time with this comment. We politely refer to our previous answers. We note that we used the correlations as criterion parametric variables (we were not interested in their significance) and thus did not perform Bonferroni correction taking the number of correlations into account.

Appendix B

Dear Dr Demski,

We are pleased with the opportunity to resubmit our manuscript titled “Twitter, Time and Emotions” to Royal Society Open Science.

We respond below to your helpful comments and those of the reviews. We hope our manuscript is now acceptable for publication.

With best wishes,
The Authors

Associate Editor Comments to Author (Dr Christina Demski):

While it is evident that some of the reviewer concerns have now been addressed, unfortunately one reviewer is not satisfied with the scientific rationale of the paper. This is a concern that has now been raised by two reviewers and means we are not able to accept the manuscript in the present form. However, if you would like to resubmit at a later stage, this option is open to you.

>> We have removed analyses on associations between LIWC dimensions and positive and negative affect and now focus exclusively on circadian and circaseptan patterns of change in emotional content and propose 3 types of comparisons: a) between Self-referencing tweets vs Other topic tweets (new dataset of 18 million tweets collected during the same time period as the initial dataset), b) between emotional coding of textual productions vs emotional coding of emojis, and finally c) between the coding of textual productions using different tools (LIWC, VADER and Hu Liu). We have thus rewritten most of the paper (now close to 12’000 words long), including an increased focus on the scientific rationale of the manuscript by providing a thorough literature review on the different topics that are relevant to the research questions addressed in the paper.

The most important differences with regards to each comparison relate to: a) differences in the amplitude of most circadian and circaseptan patterns in the comparison between Self-referencing tweets compared with Other topic tweets and some differences in pattern shape (e.g., Hu & Liu positive, circaseptan pattern), b) marked differences in the positive dimensions for the Hu & Liu in the comparison with to the other textual analysis tools, and c) the circaseptan patterns in the negative dimension in the analysis of emojis (lowest values during the week-end in both data sets), which is opposite to those observed in all textual analysis tools (highest values during the week-end) and thereby displaying the complementarity of emotional expression in emojis and text.

Reviewer comments to Author:
Reviewer: 2

Comments to the Author(s)
My issues have been addressed by the authors

>> We thank Reviewer 2 for acknowledging our work in addressing her/his constructive comments in the previous iteration of the manuscript.

Reviewer: 1

Comments to the Author(s)
Dear Authors,
your chosen topic is of real interest, but this article does not have any new message for the reader. Besides replicating known studies in slightly different settings, there is no evidence that those settings are any better, there is no discussion of lesson learnt from the differences, and the two statistical tests are not justified, badly explained, and lead to no insight. My advice is: start from what you want to find out, then work your way backwards to the data and analysis. I really wanted to learn something new and clear from those experiments, but I could not.

>> We believe Revision 1 of the manuscript successfully addressed Reviewer 1's former comments. It surprised us that a lack of relevance of the analyses that we carried out was pointed out at this stage rather that at the moment of the initial submission. We wish to emphasize that the statistical analyses (mixed-model regression) we undertook conform with the state of the art. They add value to the existing literature which did not use analyses that allowed partialling-out user-level variance (due to the nesting of tweets within participants) from observation-level variance, and therefore might have led to biased results. We still are of the opinion such analyses are necessary and have thus again relied upon mixed-model regression in the current submission.

We have now increased the theoretical foundations of our paper which now focuses entirely on circadian and circaseptan patterns of change in emotional content in tweets. We provide new analyses which we believe add supplementary value to our contribution by focusing on 3 types of comparisons in temporal patterns: a) between Self-referencing tweets vs Other topic tweets (new dataset of 18 million tweets collected during the same time period as the initial dataset), b) between emotional coding of textual productions vs emotional coding of emojis, and finally c) between the coding of textual productions using different tools (LIWC, VADER and Hu Liu).

Appendix C

Cover letter

Dear Dr Grimshaw,

We thank you for your decision on our manuscript titled "Twitter, Time and Emotions" and your very useful comments and suggestions. These have improved the contribution of our work, of which we are pleased to submit the revised version.

We briefly describe below in italics the changes we made to the manuscript, starting with your suggestions.

With best wishes,
The Authors

Editorial comments.
1. LIWC and VADER are acronyms for well-known coding systems. However, for non-expert readers it would be useful to provide the full names of these systems on first mention. Moreover, given that one goal of the manuscript is to compare systems, it would be useful to highlight how the systems differ conceptually. This could be done briefly when the measures are described in the Method, and referred to in the Discussion.

We have included more details on the conceptual differences between the mentioned coding systems and used their full name on first mention.

2. I agree that the mixed-model regression are the appropriate way to analyse the data, but one problem with the method is that it can be over-powered (particularly when used with such large samples), with the result that everything is significant. I think using the **F values for comparison** of conditions is a reasonable way to compare effects, but what of the different polynomials (linear to quintic) which are also all signficant? Table 2 provides only the F value for the complete model but not the trends, and so it is not possible to know if the trends differ in their relative importance to the overall pattern. Are the B values (provided in text) interpretable? Moreover, you then provide qualitative descriptions of the patterns (e.g., the proportion of PA might peak at midnight, decline until 8 am, and then slowly rise again...). If the descriptions are so qualitative, what do the individual polynomial trends contribute? Readers could use some guidance here on how to interpret the polynomial effects.

We have now included the F values for the individual trends, extended the results section to include comments on the relative contribution of the trends (focusing on the most important ones) in conjunction with the sign of the respective regression coefficient. Because the coefficients are differently scaled (see methods), they do not provide information with regards to the magnitude of the effects.

3. The files on OSF provide the unique identifiers, location, time, and date of each tweet. However, for the purposes of reproducing your findings, it would be useful to provide the values of each dimension according to each coding system, and the code for your statistical analyses of that data. I don't see that these data should violate any of twitter's licensing requirements.

We have now included the requested data and scripts on the OSF platform.

Other changes.
We have slightly reorganized the manuscript and improved on the writing of a few sentences.
